# Advances in Catalysts for Magnesium-Based Hydrogen Storage Materials

**DOI:** 10.34133/research.1036

**Published:** 2025-12-22

**Authors:** Yong Zhu, Wenhao Ma, Xingzai Chai, Yunpeng Gu, Wenqi Wu, Haisheng Chen, Ting Zhang

**Affiliations:** ^1^ School of Energy and Mechanical Engineering, Nanjing Normal University, Nanjing 210042, China.; ^2^ Nanjing Institute of Future Energy System, Nanjing 211135, China.; ^3^ University of Chinese Academy of Sciences, Nanjing 211135, China.; ^4^ University of Chinese Academy of Sciences, Beijing 100049, China.; ^5^ Institute of Engineering Thermophysics, Chinese Academy of Sciences, Beijing 100190, China.

## Abstract

MgH_2_ (magnesium hydride) has attracted attention as a potential hydrogen storage material owing to its rich availability and high theoretical hydrogen capacity. Nevertheless, its practical utilization is restricted by its high thermodynamic stability and slow hydrogen sorption kinetics. Recent advancements have demonstrated that incorporating various catalytic systems—such as transition metals, metal oxides, metal halides, metal sulfides, and carbon-supported materials—effectively improves hydrogen dissociation, diffusion, and Mg–H bond modulation. Structural transformations, interfacial interactions, and synergistic effects in multicomponent systems substantially enhance MgH_2_ performance. Furthermore, cutting-edge computational techniques such as DFT (density functional theory) and ML (machine learning) have become indispensable for expediting catalyst development and forecasting their performance. These computational techniques enable high-throughput screening, provide atomic-scale insights into catalytic mechanisms, and substantially reduce experimental workloads. This review systematically summarizes recent progress in catalytic modifications of MgH_2_, elucidates the underlying enhancement mechanisms, highlights the contributions of DFT and ML methodologies, and discusses future directions such as nanostructuring, multifunctional composite catalysts, and computational-driven rational catalyst design for next-generation hydrogen storage technologies.

## Introduction

Solid-state hydrogen storage has emerged as a safe and efficient alternative to conventional hydrogen storage methods [1–4]. Among diverse solid-state materials, metal hydrides stand out due to their notable advantages, including high hydrogen storage densities and reversible hydrogen absorption and desorption capacities. In particular, MgH_2_ (magnesium hydride) has gained growing attention due to its abundant natural availability and impressive theoretical hydrogen storage capacity of 7.6 wt% [5–8].

Nonetheless, the widespread use of MgH_2_ is severely limited by its substantial thermodynamic stability and slow kinetics during hydrogenation and dehydrogenation processes. For example, the hydrogenation/dehydrogenation reaction has an enthalpy of approximately 75 kJ/mol. Consequently, the desorption temperature under atmospheric pressure is around 311 °C, which far exceeds the practical range for most applications. Moreover, because magnesium lacks d orbitals, it interacts weakly with the σ* antibonding orbitals of H_2_. As a result, H_2_ dissociates slowly on the Mg surface. Additionally, surface oxidation [e.g., MgO and Mg(OH)_2_ layers] further hampers hydrogen diffusion. Effective hydrogen storage is therefore limited to elevated temperature and pressure.

To overcome these challenges, several optimization strategies, such as nanoscaling, alloying, and catalytic modification, have been proposed [9–12]. Among these strategies, catalytic modification is particularly effective. It promotes H_2_ dissociation, accelerates atomic hydrogen diffusion, and tunes the stability of Mg–H bonds, thereby improving the kinetics of MgH_2_ [13].

**Fig. 1.**
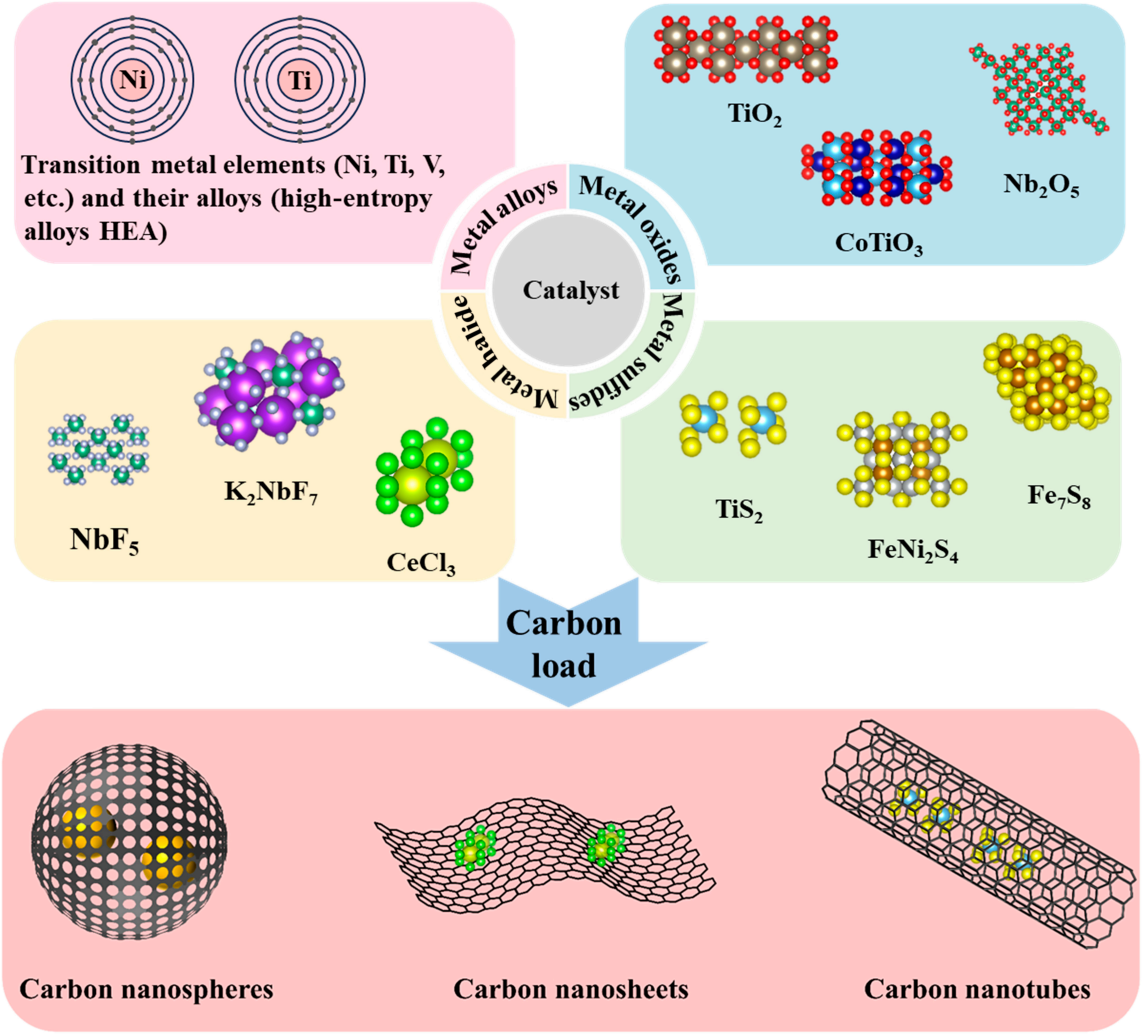
Commonly used catalyst types for MgH_2_ hydrogen storage.

A wide range of catalysts—including transition metals and their alloys, metal oxides, metal halides, metal sulfides, and carbon-supported materials—show substantial promise for improving MgH_2_ performance. Representative examples are summarized in Fig. 1. Some magnesium-based hydrogen storage materials already meet the DOE (U.S. Department of Energy) 2025 targets for hydrogen storage quality and refueling rate but remain somewhat distant in terms of cost and cycle life.

This review systematically examines recent advancements in catalytic systems applied to MgH_2_-based hydrogen storage. Initially, the intrinsic physicochemical characteristics and hydrogen storage mechanisms of MgH_2_ are described. Subsequently, we explore catalytic mechanisms associated with various catalyst types and analyze their impacts on the hydrogen storage properties. Finally, drawing on existing studies, we outline the remaining challenges and future research avenues. Our aim is to provide theoretical insight and practical guidance for advancing high-performance hydrogen storage materials.

## Catalytic Mechanism of Hydrogen Storage in MgH_2_

Hydrogen storage in MgH_2_ is a reversible solid–gas reaction: under suitable temperature and H_2_ pressure, magnesium forms the hydride (Eq. 1).

$$\mathrm{Mg}+H_{2}↔{\mathrm{MgH}}_{2}\;\;\;\;\;\;\;\Delta H=75\;\mathrm{kJ}/\mathrm{mol}$$ (1)

This exothermic reaction is reversible and can be driven in either direction by adjusting the temperature and the partial pressure of hydrogen. The mechanism proceeds in stages: (a) physisorption of H_2_ on the surface followed by dissociation; (b) chemisorption and surface diffusion of H atoms; and (c) incorporation into the Mg lattice, where MgH_2_ nucleates and grows. Conversely, dehydrogenation must overcome a substantial activation energy. Its kinetics are limited by the formation of hydrogen vacancies and the cleavage of Mg–H bonds [14].

Thermodynamically, the dehydrogenation reaction of MgH_2_ is characterized by an enthalpy change (Δ*H*) of approximately 75 kJ/mol and an entropy change (Δ*S*) of about 130 J·K^−1^·mol^−1^, as described by the van’t Hoff equation (Eq. 2):

$$\ln P=\frac{\Delta H}{\mathrm{RT}}-\frac{\Delta S}{R}$$ (2)

where 𝑃 is the equilibrium hydrogen pressure, *T* is the absolute temperature, *R* is the ideal gas constant (8.314 J·mol^−1^·K^−1^), and Δ𝐻 and Δ*S* are the enthalpy and entropy changes of the dehydrogenation reaction, respectively.

The equilibrium dehydrogenation temperature is approximately 311 °C at standard atmospheric pressure, indicating a substantial thermodynamic barrier to hydrogen desorption under conventional operating conditions [15]. To address the thermodynamic and kinetic challenges, incorporating catalysts has emerged as an essential approach to improve the hydrogen storage capability of MgH_2_.

Appropriate catalysts can greatly enhance the rates of hydrogen absorption and desorption by reducing the energy barrier for H_2_ dissociation, promoting the surface diffusion of hydrogen atoms, and adjusting the stability of Mg–H bonds [16,17]. By Sabatier’s principle, an effective catalyst should bind hydrogen moderately—strong enough to dissociate H_2_ but weak enough to avoid retaining H atoms—so that hydrogen transfers smoothly into the Mg matrix.

During the reaction, some catalysts can undergo in situ transformation into highly active intermediate phases, such as metal hydrides, alloys, or nanometal clusters. These intermediates often provide additional hydrogen diffusion pathways or reduce the binding energy of Mg–H bonds [18]. In addition, interfacial effects—such as the hydrogen spillover phenomenon and hydrogen pumping mechanism—are pivotal to both hydrogen absorption and desorption, substantially improving the overall kinetic performance. To analyze the kinetics of hydrogen absorption and desorption, models such as the JMAK (Johnson–Mehl–Avrami–Kolmogorov) model and diffusion-controlled models are commonly employed, offering insights into the rate-determining steps and catalytic pathways.

## Metal and Alloy Catalysts

The d orbitals of transition metals possess relatively high energy levels that are closely aligned with the σ* antibonding orbitals of hydrogen molecules. This energetic compatibility facilitates effective orbital interactions, which weaken the H–H bonds and subsequently lower the energy barrier for hydrogen dissociation [19]. The metal and alloy catalysts involved in this article are shown in Table S1.

**Fig. 2.**
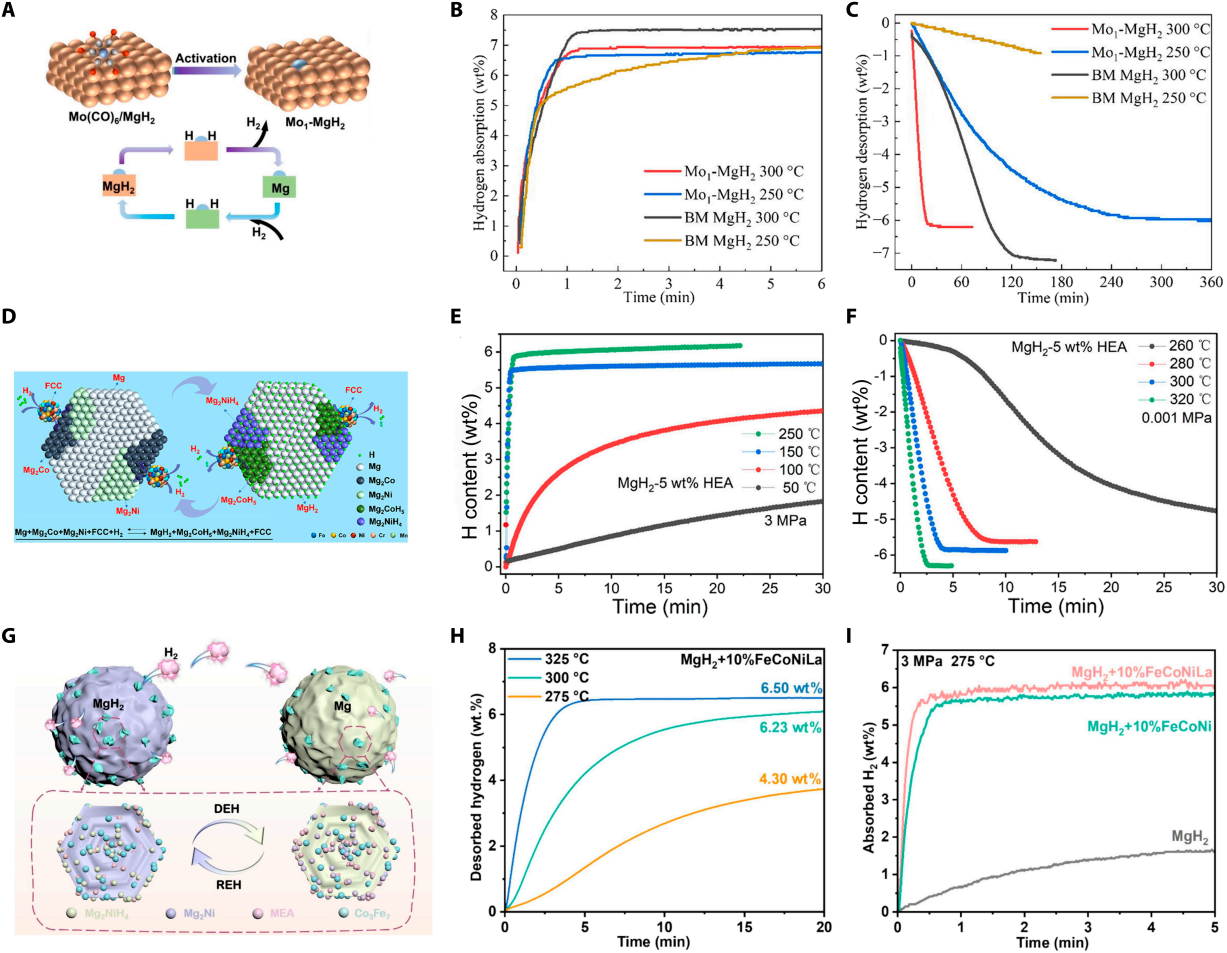
(A) Schematics of the hydrogenation and dehydrogenation mechanisms of MgH_2_ doped with Mo_1_. (B) Hydrogenation profiles and (C) corresponding dehydrogenation profiles of MgH_2_ doped with Mo_1_ at different temperatures [21]. (D) Schematics of the hydrogenation and dehydrogenation mechanisms of MgH_2_ doped with HEA. (E) Hydrogenation profiles and (F) corresponding dehydrogenation profiles of MgH_2_ doped with 5 wt% HEA at different temperatures [23]. (G) Schematics of the hydrogenation and dehydrogenation mechanisms of MgH_2_ doped with FeCoNiLa. (H) Dehydrogenation behavior of MgH_2_ doped with 10 wt% FeCoNiLa at different temperatures. (I) Hydrogenation behavior of MgH_2_ doped with 10 wt% FeCoNi and FeCoNiLa catalysts at 275 °C under 3 MPa [24].

Initial investigations into enhancing the dynamics of MgH_2_ centered on single-element catalysts. For instance, Yan et al. [20] reported that nano-Mn catalysts substantially reduced the onset dehydrogenation temperature of MgH_2_ from 355 to 175 °C and decreased the activation energy for hydrogenation from 72.5 to 18.8 kJ/mol. Furthermore, 6.7 wt% of hydrogen was desorbed within 5 min at 300 °C, with 92% capacity retention after 20 cycles. The enhancement was attributed to the stable dispersion of nano-Mn, which provided interfacial active sites and facilitated hydrogen diffusion. Nevertheless, nanoparticle agglomeration and surface oxidation during cycling remain critical barriers to long-term durability. To overcome these drawbacks, attention has shifted toward SACs (single-atom catalysts), which maximize atomic utilization and interfacial reactivity. Yang et al. [21] developed an alloy-type Mo SAC (Mo_1_-MgH_2_) by loading Mo(CO)_6_ onto MgH_2_ via impregnation and in situ activation. The synthesized Mo_1_–MgH_2_ composite demonstrated a marked decrease in the onset desorption temperature to 218 °C, along with a lowered activation energy for dehydrogenation at 110 kJ/mol. It absorbed 6.77 wt% H_2_ within 1 min (Fig. 2B) and desorbed over 5.0 wt% within 150 min at 250 °C (Fig. 2C), while maintaining nearly 100% capacity after 10 cycles. These improvements were attributed to the atomic-scale integration of Mo with MgH_2_, which enabled efficient hydrogen adsorption, dissociation, and diffusion, as well as facilitated Mg–H bond cleavage during dehydrogenation (Fig. 2A). However, robustly anchoring metals as isolated single atoms at defect or coordination sites requires stringent control of precursors, atmosphere, and temperature, which substantially elevates costs. Moreover, maintaining comparable defect densities and uniform dispersion during scale-up is difficult, thereby hindering large-scale manufacturing.

Compared with single-element catalysts, multimetal alloys offer tunable electronic structures and cooperative interfacial chemistry. Yang et al. [22] synthesized FeCo nanosheets via wet chemical methods and investigated their catalytic effect in the MgH_2_ system. The composites exhibited a reduced dehydrogenation temperature range of 200 to 320 °C and desorbed 6 wt% hydrogen in 9.5 min at 300 °C. The activation energy was lowered to 65.3 kJ/mol. The Fe–Co bimetallic system offered numerous active sites for H_2_ dissociation and facilitated hydrogen spillover onto the MgH_2_ surface, thereby greatly improving the kinetics of hydrogen storage. However, potential risks still exist regarding the compositional segregation of Fe/Co and the morphological stability of ultrathin nanosheets during cycling.

In contrast, compared with conventional binary and ternary alloys, HEAs (high-entropy alloys) and MEAs (medium-entropy alloys) offer superior structural and thermal stability, as well as enhanced catalytic activity. Wan et al. [23] demonstrated that doping MgH_2_ with 5 wt% FeCoNiCrMn HEA enabled the adsorption of 5.5 wt% hydrogen within 0.5 min at 150 °C (Fig. 2E) and the desorption of 5.8 wt% hydrogen within 4 min at 280 °C (Fig. 2F), with a reduced dehydrogenation activation energy of 90.2 kJ/mol. Furthermore, its hydrogen storage capacity remained at 98.6% after 50 cycles, far exceeding that of most similar catalysts. However, this still falls short of the cycle life specified in the 2025 DOE’s target. This improvement was mainly attributed to the synergistic catalytic effect of the multiple elements in the HEA, which facilitated both hydrogen dissociation and the rapid cleavage of Mg–H bonds (Fig. 2D). Furthermore, Jiang et al. [24] synthesized an amorphous FeCoNiLa MEA via pulsed electrodeposition and used it as a catalyst for MgH_2_. Doping MgH_2_ with 10 wt% FeCoNiLa enabled the desorption of 6.5 wt% hydrogen within 5 min at 325 °C (Fig. 2H) and reduced the dehydrogenation activation energy from 159.3 to 103.9 kJ/mol. It also facilitated rapid hydrogen absorption, with 6 wt% absorbed within 1 min at 275 °C (Fig. 2I). The composite maintained 98.2% of its initial capacity after 20 cycles. The enhanced performance was attributed to the multiphase synergistic catalysis of FeCoNiLa and in situ formed Co_3_Fe_7_, as well as the reversible Mg_2_Ni/Mg_2_NiH_4_ phase, which contributed a hydrogen pumping effect, together facilitating Mg–H bond dissociation and hydrogen diffusion (Fig. 2G). However, obtaining a near-atomic single-phase solid solution typically requires high-purity metals and energy-intensive processing, which results in manufacturing costs exceeding the DOE’s target of $9/kWh.

To reduce production costs, some research has turned to economical and environmentally friendly synthetic methods, aiming to achieve similar kinetics with simpler chemical reactions and lower processing burden. Huang et al. [25] developed a mechanochemical synthesis method for highly dispersed Ni–N–C composites using simple ball milling, pyrolysis, and aqueous washing, without relying on large quantities of organic solvents. When applied to MgH_2_, the catalyst facilitated hydrogen desorption of 5.4 wt% within 5 min at 300 °C and lowered the activation energy for dehydrogenation to 87.2 kJ/mol. This improvement was mainly ascribed to the highly electronegative sites within the Ni–N–C framework, which effectively enhanced hydrogen molecule dissociation. In addition, Machmud and Jalil [26] prepared Ti-doped MgH_2_ composites using HPRM (high-pressure reactive ball milling). The introduction of Ti nanoparticles effectively suppressed Mg grain growth and substantially lowered the activation energies for both hydrogenation and dehydrogenation, with complete dehydrogenation achieved in just 6 min at 350 °C. Similarly, Rahwanto et al. [27] synthesized MgH_2_ composites doped with 2 mol% Ni nanoparticles via high-pressure ball milling. Under 100 bar of hydrogen at 300 °C, these composites achieved 5.3 wt% hydrogen absorption within 5 min and completed hydrogen desorption within only 4 min at 50 mbar, demonstrating outstanding sorption kinetics.

## Metal Oxide Catalysts

Compared with traditional metal-alloy catalysts, metal oxides offer superior thermal stability, better particle dispersion, and cost-effective synthesis—making them well suited to MgH_2_-based hydrogen storage. The metal oxide catalysts involved in this article are shown in Table S2.

Using TiO_2_ as an early model system, accumulating evidence shows that metal oxide activity depends strongly on crystal structure and morphology. Pereira et al. [28] tailored TiO_2_ nanocatalysts (NR550/NR650/NR750) by calcining titanate nanotubes at 550/650/750 °C. Among them, NR750 contains a small amount of rutile. Under identical Sieverts conditions (350 °C; 10 bar H_2_ absorption/0.1 bar desorption), NR750-doped MgH_2_ reached 72% of its maximum absorption within 5 min, outperforming NR550 and NR650. In desorption, all samples desorbed 90% to 100% H_2_ in 3 min, with NR750 hitting this benchmark earliest. Rutile TiO_2_ is highly lattice-compatible with β-MgH_2_ and can form a tight MgH_2_–TiO_2_ interface, enabling more efficient interfacial hydrogen exchange. Moreover, Ma et al. [29] synthesized TiO_2_ nanosheets (TF10/TF30/TF50/TF70) with tunable 001 facet exposure via a hydrothermal method. Wulff estimates give 001 fractions of 20/34/59/88%. At 5 wt% TiO_2_, the 001-rich TF70 showed the fastest kinetics, achieving 5.3 wt% hydrogen absorption in 44 s at 200 °C and 6.4 wt% hydrogen desorption in 700 s at 300 °C. The dehydrogenation onset decreased to about 220 °C and the apparent activation energy decreased to 76 kJ mol^−1^. Mechanistically, the major 001 facets fully expose the pentacoordinated Ti and have higher surface energy than 101, providing more active sites for H_2_ dissociation/recombination.

**Fig. 3.**
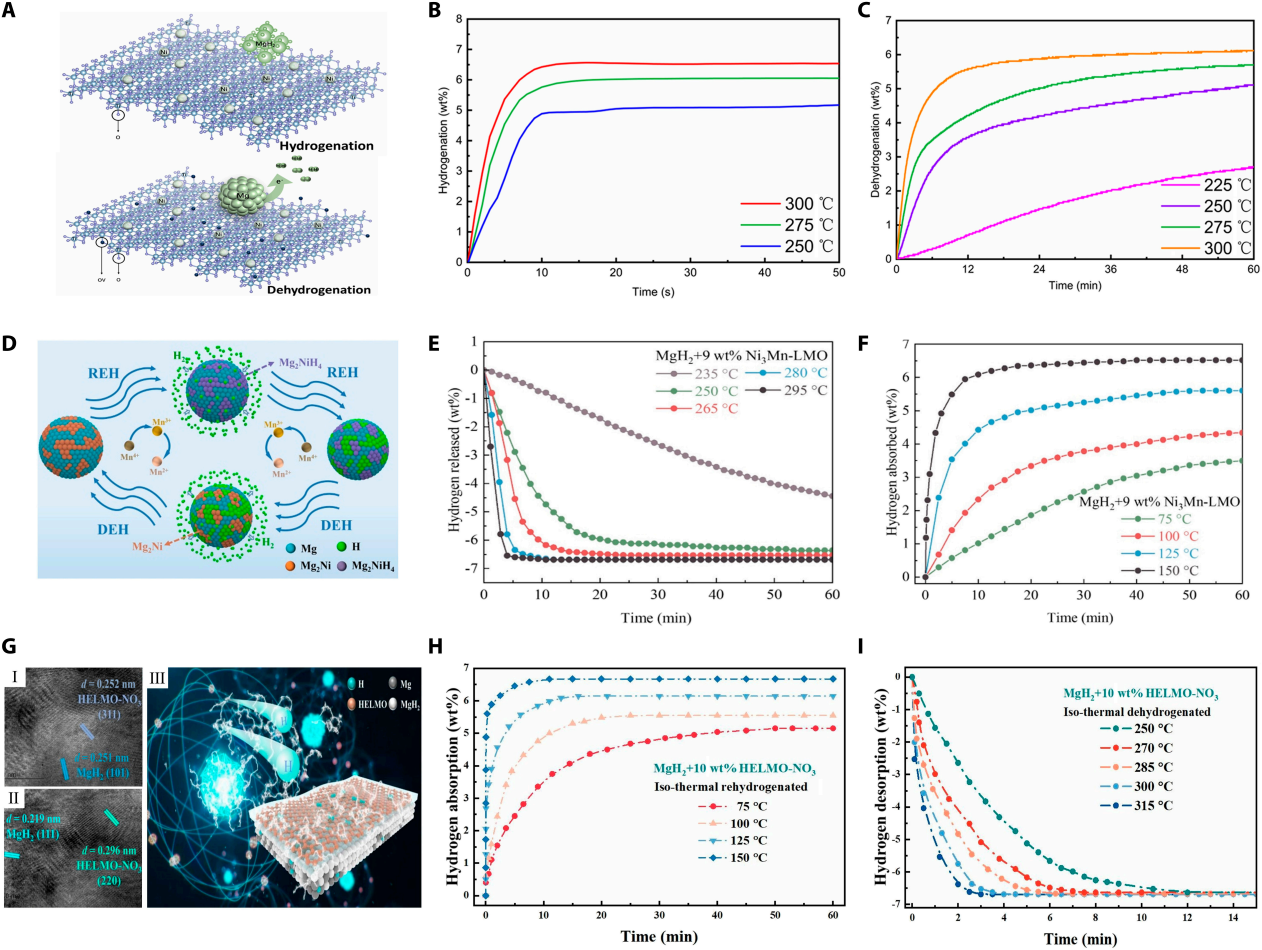
(A) Schematics of the hydrogenation and dehydrogenation mechanisms of MgH_2_ doped with Ni_0.034_@TiO_2_. (B) Hydrogenation profiles and (C) corresponding dehydrogenation profiles of MgH_2_ doped with 15 wt% Ni_0.034_@TiO_2_ catalyst at different temperatures [30]. Reproduced with permission [30]. Copyright 2025, American Chemical Society. (D) Schematics of the hydrogenation and dehydrogenation mechanisms of MgH_2_ doped with Ni_3_Mn-LMO. (E) Dehydrogenation profiles and (F) corresponding hydrogenation profiles of MgH_2_ doped with 9 wt% Ni_3_Mn-LMO catalyst at different temperatures [34]. (G) HRTEM images (I and II) of the ball-milled MgH_2_ + 10 wt% HELMO-NO_3_. (III) Schematics of the hydrogenation and dehydrogenation mechanisms of MgH_2_ doped with HELMO-NO_3_. (H) Hydrogenation profiles and (I) corresponding dehydrogenation profiles of MgH_2_ doped with 10 wt% HELMO-NO_3_ catalyst at different temperatures [35].

On the basis of morphology control, methods such as single-atom anchoring and heterogeneous element doping can further reshape the interface energy band and the electronic structure of the active site to obtain better catalytic effects. Zhang et al. [30] synthesized a TiO_2_-supported SAC (Ni_0.034_@TiO_2_) via a molten salt approach. This catalyst markedly lowered MgH_2_’s initial hydrogen desorption temperature to 200 °C, achieving hydrogen desorption of 4.6 wt% within 5 min at 300 °C (Fig. 3C). Notably, rapid hydrogen absorption of 6.53 wt% occurred within 10 s (Fig. 3B). Kinetic analysis indicated a substantial decrease in activation energies to 64.35 kJ/mol for desorption and 35.17 kJ/mol for hydrogen absorption. Moreover, the catalyst exhibited excellent cycling stability, retaining 97.26% hydrogen capacity after 100 cycles. These improvements were attributed to synergistic effects from atomically dispersed Ni, abundant oxygen vacancies, and multivalent Ti species, collectively facilitating electron transfer and reducing Mg–H bond strength, thereby enhancing sorption kinetics. The single-atom dispersion also maximized atom utilization and thermal stability (Fig. 3A). Moreover, Wang et al. [31] investigated nitrogen-doped Nb_2_O_5_, demonstrating full hydrogenation at 70 °C under 50 atm hydrogen pressure and rapid hydrogen absorption of 5.1 wt% at 100 °C within 3 min. The improved performance resulted from cooperative effects among Nb–N–O clusters, promoting hydrogen diffusion and Mg–H bond cleavage.

Compared with single-cation oxides, polymetallic oxides can further regulate hydrogen adsorption, enrich oxygen vacancies, and shorten diffusion paths by generating heterogeneous interfaces and complementary redox pairs. Liang et al. [32] synthesized NiV_2_O_6_ using a simple hydrothermal process, showing that MgH_2_ doped with 10 wt% NiV_2_O_6_ substantially improved hydrogen sorption performance at 150 °C, absorbing 5.59 wt% hydrogen in 50 min. In addition, NiV_2_O_6_ reduced the hydrogen desorption activation energy from 119.2 to 92.9 kJ/mol, allowing 5.2 wt% hydrogen to be rapidly desorbed within 10 min at 300 °C. To further improve the catalytic performance, Zhang et al. [33] loaded the bimetallic oxide CoMoO_4_ on rGO (reduced graphene oxide). Experiments showed that MgH_2_ with the addition of 10 wt% CoMoO_4_/rGO could absorb 4.2 wt% of hydrogen within 20 min at 150 °C and desorbed 6.25 wt% of hydrogen within 10 min at 300 °C. rGO incorporation stabilized the nanostructure and increased exposed active sites, facilitating hydrogen diffusion and reducing Mg–H bond strengths, thereby optimizing storage kinetics.

Recent studies have shown a growing trend toward 2-dimensional layered multimetal oxide catalysts, especially those with synergistic effects of cations and anions. Zhong et al. [34] synthesized 2-dimensional Ni_3_Mn-based layered metal oxide (Ni_3_Mn–LMO) catalysts through hydrothermal synthesis and annealing. MgH_2_ composites doped with 9 wt% Ni_3_Mn–LMO showed markedly reduced hydrogen desorption temperatures (190 °C) and activation energies (86.9 kJ/mol). This system desorbed 6.59 wt% hydrogen within 5 min at 295 °C (Fig. 3E) and rapidly absorbed hydrogen (6.1 wt% within 600 s at 150 °C, and 3.49 wt% within 60 min at 75 °C) (Fig. 3F). Enhanced performance resulted from the formation of Mg_2_Ni/Mg_2_NiH_4_ phases and electron transfer facilitated by multivalent Mn species (Fig. 3D). The layered nanosheet structures also contributed high surface areas and abundant catalytic sites. Further innovation was reported by Wang et al. [35], who synthesized 2-dimensional HELMO-NO_3_ catalysts via hydrothermal processing of Cr, Mn, Fe, Co, and Ni nitrates, followed by calcination at 500 °C under argon. With 10 wt% HELMO-NO_3_ doping, MgH_2_ exhibited a lower onset desorption temperature of 184.1 °C, desorbed 6.68 wt% hydrogen in 3 min at 315 °C (Fig. 3I), and achieved a hydrogen absorption of 5.15 wt% within 50 min at 75 °C (Fig. 3H). The exceptional performance arose from a combination of high-entropy effects, lattice distortion, multielement catalytic synergy, and an ultrathin layered structure providing ample surface areas, active sites, and diffusion channels (Fig. 3G). However, the synthesis process of this type of catalyst is complex and highly sensitive to process fluctuations, which limits its large-scale application.

## Metal Halide Catalysts

Unlike metal oxides, halide catalysts (e.g., CeCl_3_ and K_2_NbF_7_) exhibit strong Lewis acidity, enabling them to directly weaken the Mg–H bond strength. Additionally, these halides often react with MgH_2_ to generate in situ nanophases (e.g., MgF_2_ and KMgH_3_). These phases shorten hydrogen diffusion paths and enhance storage kinetics. The metal halide catalysts involved in this article are shown in Table S3.

Metal chloride is one of the earliest and most widely used catalysts of this type, with low cost, wide source, and mature technology. In the field of transition metals, Ismail [36] investigated HfCl_4_ doping, reporting a notable decrease in initial hydrogen desorption temperature to 265 °C, accompanied by a considerable drop in activation energy from 167 to 102 kJ/mol, thus markedly accelerating hydrogen desorption. Similarly, Suárez-Alcántara et al. [37] explored Mg–15 wt% VCl_3_ composites and achieved a hydrogenation onset temperature as low as 50 °C, with an activation barrier of 63.8 kJ/mol. This composite absorbed 5 wt% hydrogen within just 1 min at 300 °C, maintaining a stable 6 wt% capacity after cycling. These advancements were mainly attributed to the refined Mg structure produced by cryo-ball milling and uniform VCl_3_ dispersion, which collectively improved hydrogen diffusion and interfaces. In the field of rare earth metals, Ismail [38] observed that LaCl_3_ doping led to a Mg_3_La alloy, markedly lowering the activation energy for dehydrogenation from 166 to 143 kJ/mol. The resulting composite rapidly absorbed 5.1 wt% hydrogen within 2 min at 300 °C, surpassing pure MgH_2_ (3.8 wt%). Subsequently, the group demonstrated that CeCl_3_ promoted Ce–Mg alloy formation and CeH_2.73_ during cycling, facilitating hydrogen diffusion and reducing activation energy from 167 to 149 kJ/mol. The CeCl_3_-doped composite consequently desorbed 5.5 wt% hydrogen in 10 min at 350 °C, markedly outperforming pure MgH_2_ (3.1 wt%) [39]. However, metal chlorides generate inert MgCl_2_ during catalysis; this species accumulates on the surface and blocks diffusion and interfaces. With the accumulation of cycles, the catalytic activity will gradually decrease and the apparent activation energy will gradually increase.

**Fig. 4.**
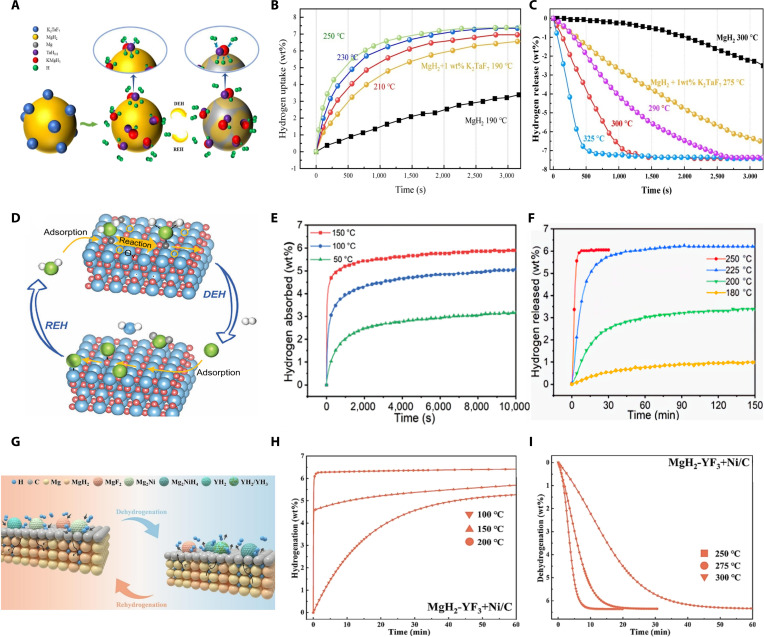
(A) Schematics of the hydrogenation and dehydrogenation mechanisms of MgH_2_ doped with K_2_TaF_7_. (B) Hydrogenation profiles and (C) corresponding dehydrogenation profiles of MgH_2_ doped with 1 wt% K_2_TaF_7_ catalyst at different temperatures [41]. (D) Schematics of the hydrogenation and dehydrogenation mechanisms of MgH_2_ doped with TiO_2−*x*_(F). (E) Hydrogenation profiles and (F) corresponding dehydrogenation profiles of MgH_2_ doped with TiO_2−*x*_(F) catalyst at different temperatures [44]. (G) Schematics of the hydrogenation and dehydrogenation mechanisms of MgH_2_ doped with YF_3_ and Ni/C. (H) Hydrogenation profiles and (I) corresponding dehydrogenation profiles of MgH_2_ doped with YF_3_+Ni/C catalyst at different temperatures [45].

Compared to chloride systems, metal fluoride catalysts exhibit stronger Lewis acidity and greater potential for interface engineering in the Mg/MgH_2_ system. During reaction, in situ MgF_2_ replaces the MgO passive layer, creating a fluorinated active surface and a nanoscale heterogeneous interface. This facilitates H_2_ dissociation/association and interfacial diffusion. Yahya et al. [40] reported that 5 wt% K_2_NbF_7_ doping reduced MgH_2_’s hydrogen desorption activation energy from 135.3 to 96.3 kJ/mol, enabling rapid desorption of 5.2 wt% hydrogen within 5.6 min at 320 °C. Notably, this composite absorbed 4.7 wt% hydrogen in 30 min at 150 °C, markedly superior to ball-milled MgH_2_ (0.7 wt%). Yan et al. [41] synthesized K_2_TaF_7_, demonstrating full hydrogen desorption (7.33 wt%) at 290 °C in 50 min (Fig. 4C). At 190 °C, the composite rapidly absorbed 6.56 wt% hydrogen, compared to only 3.45 wt% by unmodified MgH_2_ (Fig. 4B). Structural analyses identified KMgH_3_ and TaH_0.8_ phases, acting as “hydrogen pumps”, enhancing hydrogen transfer during cycling (Fig. 4A). Additionally, Yusnizam et al. [42] explored BaCoF_4_ as an innovative catalyst. MgH_2_ doped with BaCoF_4_ showed markedly decreased hydrogen desorption onset temperature (265 °C), rapidly releasing 5.0 wt% hydrogen in 30 min at 300 °C—far exceeding undoped MgH_2_ (0.1 wt%). The composite also rapidly absorbed 6.4 wt% hydrogen within just 5 min. The improved performance resulted from in situ generated CoF_3_ and Ba-containing species, lowering activation barriers and promoting hydrogen diffusion. However, metal fluorides face practical drawbacks: poor thermal stability at high temperatures and susceptibility to deliquescence, both of which reduce catalytic activity. Furthermore, their synthesis and use rely heavily on an anhydrous environment and corrosion-resistant equipment. They are susceptible to hydrolysis upon exposure to moisture, generating highly toxic HF, substantially increasing safety and environmental protection costs.

The latest research focuses on achieving multiple synergies and functional integration in material structure design and catalytic mechanisms. By introducing nano-confinement, surface defect engineering, and porous supports, the activation and diffusion of hydrogen, as well as its reversible absorption and desorption, are further enhanced. Zhu et al. [43] combined NbF_5_ with pBN (porous boron nitride), leveraging nano-confinement effects, greatly reducing MgH_2_’s hydrogen desorption temperature by 140 °C and ensuring robust cycling stability. Shi et al. [44] synthesized fluorinated TiO_2_ nanosheets [TiO_2−*x*_(F)] via hydrothermal methods and NaBH_4_ reduction, introducing abundant oxygen vacancies. The resulting catalysts reduced initial hydrogen desorption temperature to 189 °C, enabling rapid desorption of 6.0 wt% hydrogen within 6 min at 250 °C (Fig. 4F), and maintaining 93.3% capacity after 10 cycles. Additionally, the composite demonstrated superior hydrogen absorption, absorbing 6.79 wt% at 150 °C and even 3.0 wt% within 120 min at temperatures as low as 50 °C (Fig. 4E). The enhanced performance stemmed from oxygen vacancies, optimized hydrogen dissociation pathways, and reactive species (MgF_2_, hydrogen-rich solid solutions) formed in situ (Fig. 4D). Recently, Zhang et al. [45] incorporated YF_3_ and bio-derived porous Ni/C into MgH_2_ via short-duration ball milling, forming a well-dispersed dual-catalyst composite. The MgH_2_–10 wt% (YF_3_+Ni/C) sample exhibited lowered initial desorption temperature (221.1 °C), releasing 5.22 wt% hydrogen in 5 min at 300 °C and 5.74 wt% within 30 min at 250 °C (Fig. 4I), with rapid reabsorption (5.93 wt%) within 15 s at 200 °C (Fig. 4H). Capacity retention reached 98.4% after 30 cycles at 300 °C. These improvements arise from reversible YH_2_/YH_3_ and Mg_2_Ni/Mg_2_NiH_4_ “hydrogen-pump” mechanisms, plus abundant Ni nanoparticles dispersed in defect-rich carbon. Together, they enhance H_2_ dissociation and diffusion kinetics while limiting particle growth (Fig. 4G).

## Metal Sulfide Catalysts

Compared to metal halides, metal sulfide catalysts exhibit excellent resistance to hydrolysis and retain structural stability at elevated temperatures (up to 500 °C). The metal sulfide catalysts involved in this article are shown in Table S4.

The catalytic activity can be effectively improved by regulating the micromorphology of the catalyst. Layered structures can be exfoliated into 2D nanosheets with high specific surface area. This exposes many active sites and accelerates in-plane electron/ion transport, yielding higher catalytic activity. Wang et al. [3] systematically assessed various sulfides (TiS_2_, NbS_2_, MoS_2_, MnS, CoS_2_, and CuS) as MgH_2_ catalysts, identifying TiS_2_ with a layered structure as particularly effective. It substantially decreased the initial hydrogen desorption temperature by 126 °C, lowering the activation barrier to 50.8 kJ/mol. In addition to structure, the control of particle size is also an effective means to improve catalytic activity. Cheng et al. [46] synthesized a MgH_2_–Fe_7_S_8_ composite by ball milling industrial MgH_2_ and 16.7 wt% nano-Fe_7_S_8_. At 350 °C, the composite desorbed 4.40 wt% H_2_ within 30 min, markedly higher than the 2.48 wt% of pure MgH_2_. Furthermore, at 200 °C and 3 MPa H_2_, the composite absorbed 4.00 wt% H_2_ within 30 min, surpassing the 1.85 wt% of pure Mg. Fe_7_S_8_ doping reduced the apparent dehydrogenation activation energy from 165.9 kJ/mol to 130.0 kJ/mol. Kinetic analysis showed a shift from interface-controlled to diffusion-controlled dehydrogenation. In situ Fe and Mg_2_FeH_6_ formed during cycling, promoting Mg–H bond cleavage and hydrogen diffusion.

Recent research has turned to constructing catalysts with hierarchical or composite structures to provide more reactive sites. Xie et al. [47] prepared layered walnut shell NiS@NTA by hydrothermal carbonization. Doping 5 wt% NiS@NTA with Mg resulted in the composite absorbing 3.0 wt% H_2_ in 60 min at 80 °C and releasing 3.5 wt% H_2_ in 60 min at 300 °C, while simultaneously lowering the onset dehydrogenation temperature to 235 °C. The composite retained 97% of its capacity after 20 cycles. This performance improvement was attributed to the carefully designed layered walnut shell microstructure, which provided abundant interfaces and catalytically active sites. This morphology facilitated the in situ formation of MgS and Mg_2_Ni, thereby promoting electron transfer and hydrogen diffusion. However, morphology control alone offers limited gains; additional strategies are usually required to further boost catalytic performance.

**Fig. 5.**
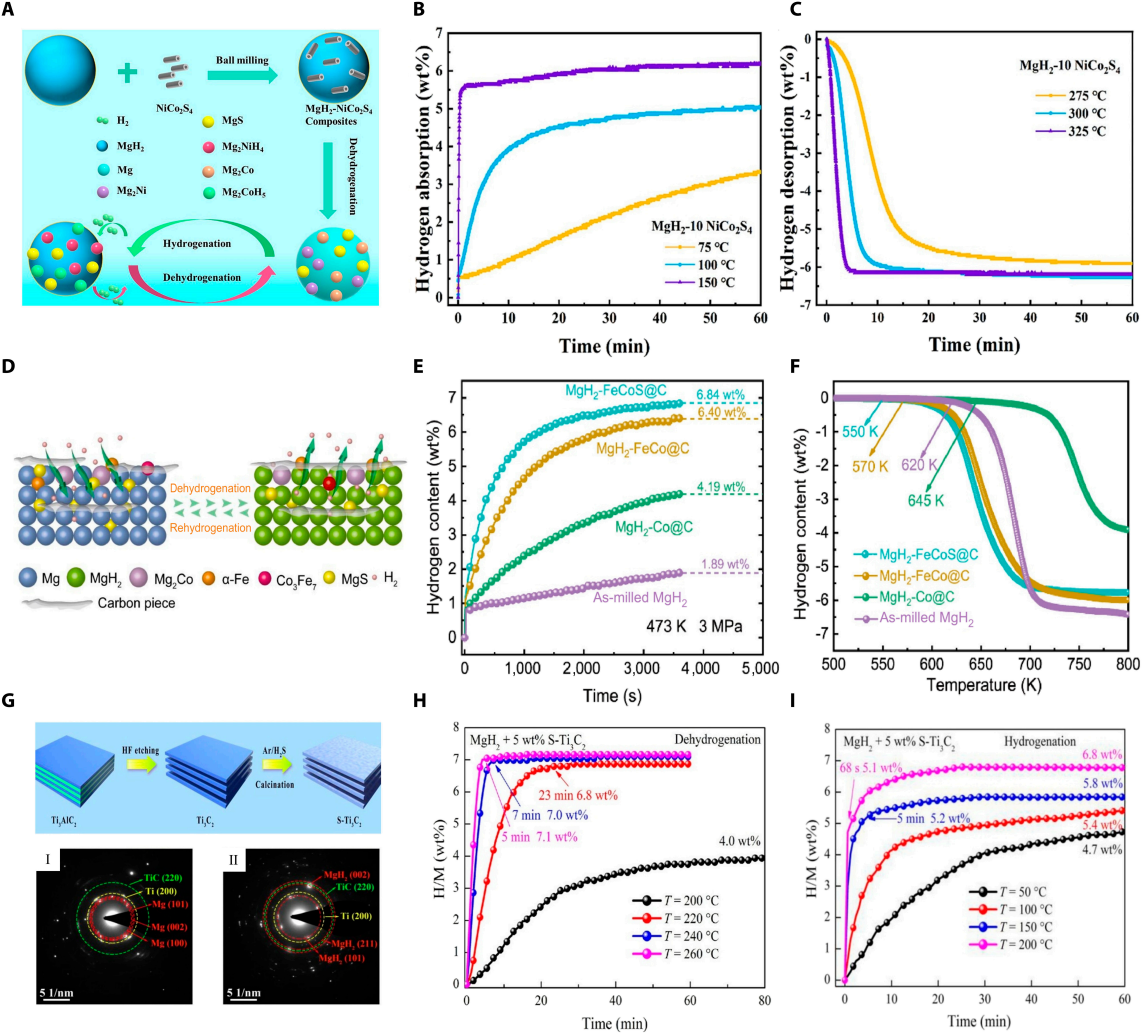
(A) Schematics of the hydrogenation and dehydrogenation mechanisms of MgH_2_ doped with NiCo_2_S_4_. (B) Hydrogenation profiles and (C) corresponding dehydrogenation profiles of MgH_2_ doped with 10 wt% NiCo_2_S_4_ at different temperatures [49]. (D) Schematics of the hydrogenation and dehydrogenation mechanisms of MgH_2_ doped with FeCoS@C. (E) Hydrogenation profiles of MgH_2_ doped with catalysts of different components at 3 MPa and 473 K. (F) TPD curves of the MgH_2_ doped with catalysts of different components [50]. (G) Synthesis scheme of S-Ti_3_C_2_ and selected-area electron diffraction (SAED) patterns for hydrogenated (I) and dehydrogenated (II) MgH_2_ containing 5 wt% S- Ti_3_C_2_. (H) Hydrogenation profiles and (I) corresponding dehydrogenation profiles of MgH_2_ doped with 5 wt% S-Ti_3_C_2_ catalyst at different temperatures [52].

Because different metal sites have complementary electronic structures and functions, multimetallic catalysts can, under close contact and in situ reconstruction, form mass-transfer interfaces with lower energy barriers. These interfaces deliver superadditive, synergistic interfacial catalysis. Tan et al. [48] synthesized a Mg-Ni-TiS_2_ nanocomposite via a solution method, in which Mg and Ni nanoparticles were in situ reduced and highly dispersed on TiS_2_ nanosheets. At 300 °C, the composite completely absorbed hydrogen within 60 s and desorbed 4.60 wt% H_2_ within 240 s, markedly outperforming pure Mg. Mechanistic studies show that in situ-formed Mg_2_NiH_4_ and TiH_2_ create fast hydrogen-diffusion pathways and lower interfacial reaction barriers. Meanwhile, MgS and TiS_2_ anchor the catalytic nanoparticles and inhibit grain growth. The advancement of bimetallic sulfide catalysts has further elevated MgH_2_-based storage performance. Chen et al. [49] developed NiCo_2_S_4_ doping (10 wt%), markedly lowering the onset hydrogen desorption temperature to 203 °C and achieving 6.11 wt% hydrogen desorption within 5 min at 325 °C, with activation energy decreased to 86.5 kJ/mol (Fig. 5C). Furthermore, hydrogen absorption reached 5.54 wt% within 1 min at 150 °C, greatly surpassing undoped MgH_2_ (2.04 wt% within 10 min) (Fig. 5B). This enhanced performance arose from the formation of Mg_2_Ni, Mg_2_Co, and MgS phases, collectively facilitating hydrogen dissociation and diffusion and enabling a reversible hydrogen pumping effect (Fig. 5A). Fu et al. [7] additionally synthesized FeNi_2_S_4_, reporting that MgH_2_ doped with FeNi_2_S_4_ decreased the hydrogen desorption activation energy from 79.8 to 65.5 kJ/mol, markedly boosting hydrogen cycling stability and kinetics.

Some materials with highly designable porous structures and extremely high specific surface areas, such as MOFs (metal–organic frameworks) and MXenes, can be used as sacrificial templates to prepare catalysts with excellent catalytic activity. Fu et al. [50] synthesized FeCoS@C through carbonization and sulfurization of MOF precursors. With 5 wt% doping into MgH_2_, the resulting composite rapidly absorbed 6.84 wt% hydrogen within 60 min at 200 °C (Fig. 5E). Additionally, the onset desorption temperature decreased markedly to 277 °C, highlighting improved kinetics (Fig. 5F). The enhancement arises from a multiphase system—Mg_2_Co, α-Fe, and Co_3_Fe_7_—formed by in situ decomposition of FeCoS@C. These phases facilitate hydrogen diffusion via a “hydrogen channeling” effect. Moreover, MgS formation further improved structural and catalytic stability (Fig. 5D). Zheng et al. [51] similarly prepared FeNi_3_–S catalysts from NiFe-MOFs. MgH_2_ doped with 10 wt% FeNi_3_–S showed a decreased initial hydrogen desorption temperature (202 °C), 123 °C below pristine MgH_2_, releasing 6.57 wt% hydrogen in 1,000 s versus 2.0 wt% for undoped MgH_2_. This improvement was attributed to synergistic hydrogen pathways involving hydrogen pumping (Mg_2_Ni/Mg_2_NiH_4_) and enhanced diffusion at Fe/MgS interfaces. Similarly, Yuan et al. [52] fabricated S–Ti_3_C_2_ via a 2-step etching and thiourea treatment (Fig. 5G). Incorporating 5 wt% S–Ti_3_C_2_ into MgH_2_ markedly enhanced hydrogen storage, reducing the initial desorption temperature to 173 °C and enabling rapid desorption of 7.0 wt% hydrogen within 7 min at 240 °C (Fig. 5H). For hydrogen absorption, the composite absorbed 5.1 wt% hydrogen in only 68 s at 200 °C and still managed 4.7 wt% within 60 min at 50 °C (Fig. 5I). Performance enhancements were mainly due to combined sulfur doping and multivalent Ti species, forming Ti^0^/Ti^2+^ active sites (Fig. 5G), promoting Mg–H bond cleavage, and improving interface stability during cycling.

## Carbon-Based Composite Catalysts

Carbon-based composites have emerged as a research hotspot in the field of MgH_2_ hydrogen storage catalysis. These materials integrate the high catalytic activity of traditional catalysts—such as metals and metal oxides—with the inherent advantages of carbon materials, including excellent chemical stability, high specific surface area, and good electrical conductivity. This combination substantially enhances the adsorption, dissociation, and diffusion of hydrogen molecules. Additionally, a carbon coating acts as a protective layer, suppressing particle agglomeration and sintering. As a result, it improves particle dispersion and cycling stability. Consequently, carbon-supported composites offer a compelling approach to boost both the efficiency and longevity of MgH_2_-based hydrogen storage systems.

### One-dimensional carbon material

One-dimensional carbon materials focus on building a continuous “transport network” for hydrogen storage reactions. They form thermally and electrically conductive percolation pathways with low thresholds. These pathways markedly alleviate thermal hysteresis and charge-transfer bottlenecks during hydrogen absorption and desorption. Furthermore, tube cavities or grooves provide linear confinement that uniformly distributes MgH_2_ and promoter metals at the nanoscale. This shortens diffusion paths and inhibits aggregation and particle growth. Relevant one-dimensional carbon-based catalysts studied herein are summarized in Table S5.

CNTs (carbon nanotubes), with their dual-channel structure of a hollow lumen and outer wall, combine efficient hydrogen diffusion with confinement. Compared to other one-dimensional carbon materials, CNTs’ continuous inner lumen and more controllable conductive network offer advantages in low-temperature dynamics and cycle life. Liu et al. [53] synthesized BCNTs (bamboo-shaped CNTs) encapsulating MgH_2_, achieving rapid hydrogen absorption of 5.79 wt% within 5 min at 250 °C by restricting MgH_2_ particle sizes to 15 to 20 nm. The activation energy for hydrogen desorption was markedly decreased from 209.18 to 97.94 kJ/mol, indicating that BCNTs are highly effective for high-density hydrogen storage. Duan et al. [54] prepared Ni-CNTs via hydrothermal and calcination. MgH_2_ with 5 wt% Ni-CNT addition desorbed 7.29 wt% H_2_ within 60 min at 250 °C, reducing the apparent dehydrogenation activation energy to 74.8 kJ mol^−1^. Furthermore, 7.2 wt% H_2_ was rapidly adsorbed within 30 min at 200 °C. Meanwhile, the capacity of approximately 7.2 wt% was maintained over 10 cycles. This enhanced performance stems from the nanoconfinement effect of the CNTs and the catalytic activity of interfacial Ni (10 to 12 nm metallic Ni nanoparticles uniformly dispersed on 30 nm CNTs), which effectively promotes the dissociation/recombination and diffusion of H_2_.

Through interface engineering, a CNT-based multicomponent system can establish a cascade pathway—dissociation → spillover → diffusion → hydrogen pump—and a stable confined microenvironment. This combination lowers the initial desorption temperature and apparent activation energy, and it markedly improves cycle stability and rate performance. Lu et al. [55] synthesized a composite catalyst comprising TiO_2_–ZnTiO_3_ on CNTs, which reduced the initial hydrogen desorption temperature from 345 to 197 °C and decreased the activation energy by 42 kJ/mol. The resulting composite delivered 6.1 wt% H_2_ in 10 min at 300 °C, with 97% capacity retention after 20 cycles. The enhanced performance resulted from the cooperative interaction of CNTs and the formation of active phases such as TiO_2_, MgTi_2_O_4_, and Zn_4_TiO_6_. Fu et al. [56] fabricated NiFe@CNT catalysts, which effectively decreased the initial MgH_2_ desorption temperature to 225 °C and lowered the activation energy to 49.7 kJ/mol. This performance improvement originated from the combined hydrogen pump function of Mg_2_Ni/Mg_2_NiH_4_ and hydrogen diffusion channels from α-Fe. Wan et al. [57] developed a VTiFe-CNTs dual-phase catalyst, lowering the dehydrogenation temperature of MgH_2_ to 200 °C and facilitating rapid hydrogen desorption of 6.3 wt% in 5 min, with 97.3% capacity retention upon cycling. The improvement was linked to abundant active catalytic sites formed by α-Fe and FCC phases, as well as the confinement effects of CNT structures. Similarly, Duan et al. [58] synthesized a bimetallic Co@Pd-CNT catalyst, achieving rapid hydrogen desorption of 7.30 wt% in 8 min at 325 °C, without noticeable performance decay after 10 cycles. This superior catalytic activity was due to dual-catalytic mechanisms involving hydrogen pumping and spillover effects. However, the relatively high price of CNTs severely limits their practical application.

**Fig. 6.**
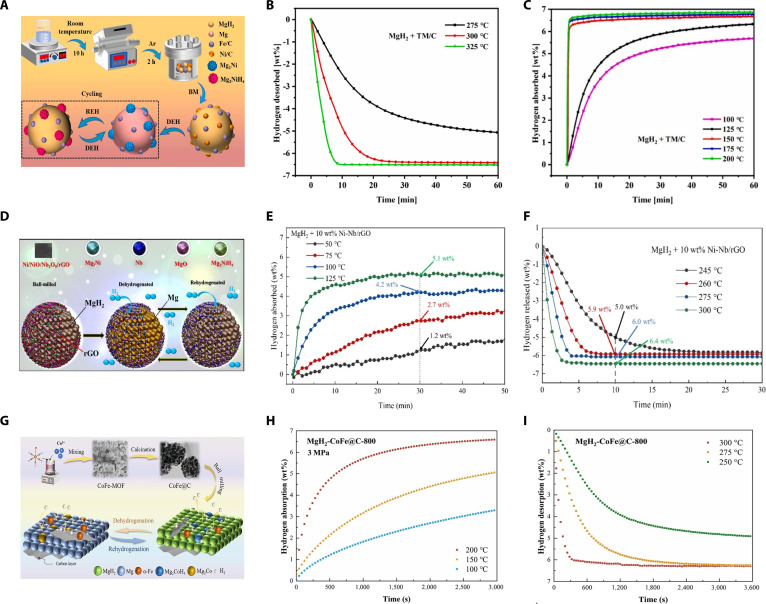
(A) Schematics of the hydrogenation and dehydrogenation mechanisms of MgH_2_ doped with TM/C. (B) Dehydrogenation profiles and (C) corresponding hydrogenation profiles of MgH_2_ doped with TM/C catalyst at different temperatures [60]. (D) Schematics of the hydrogenation and dehydrogenation mechanisms of MgH_2_ doped with Ni-Nb/rGO. (E) Hydrogenation profiles and (F) corresponding dehydrogenation profiles of MgH_2_ doped with 10 wt% Ni-Nb/rGO catalyst at different temperatures [64]. (G) Schematics of the hydrogenation and dehydrogenation mechanisms of MgH_2_ doped with CoFe@C-800. (H) Hydrogenation profiles and (I) corresponding dehydrogenation profiles of MgH_2_ doped with 10 wt% CoFe@C-800 catalyst at different temperatures [76].

One-dimensional carbon materials prepared by MOF derivatization are relatively low in cost and can achieve a certain balance between performance and cost, making them a research hotspot in recent years. Ren et al. [59] fabricated MgH_2_/Ni@pCNF composites by supporting Ni nanoparticles on MOF-derived N-doped pCNFs (porous carbon nanofibers). The composites demonstrated a reduced activation energy of 96.58 kJ/mol for hydrogen desorption, a lower initial desorption temperature of 200 °C, and retained 95.4% capacity after 10 cycles at 300 °C. Hu et al. [60] synthesized TM/C catalysts (TM = Fe, Ni) using MOF-derived porous carbon (Fig. 6A). MgH_2_ modified with 10 wt% TM/C exhibited a markedly lowered initial hydrogen desorption temperature of 194 °C, with a reduced activation energy of 77.3 kJ/mol, releasing approximately 6.5 wt% H_2_ below 300 °C (Fig. 6B). Then, the composite achieved rapid absorption of 6.2 wt% within 60 s at 150 °C and 5.7 wt% within 60 min at 100 °C (Fig. 6C). These enhancements were driven by the combined catalytic activities of Fe and Ni: Fe facilitated hydrogen dissociation, whereas Ni enhanced the reversible formation of Mg_2_Ni/Mg_2_NiH_4_ phases, resulting in effective hydrogen transport. Additionally, the carbon matrix structure prevented particle agglomeration and provided pathways for hydrogen diffusion (Fig. 6A).

### Two-dimensional carbon materials

Two-dimensional carbon materials focus on the construction and regulation of “interfaces and sites”. Their ultra-high surface area and wrinkled structure provide uniform and secure anchoring for nanocatalytic particles. Their “thin blanket” coating and planar confinement enable high-density active interfaces even at low loadings. The abundant vacancy defects also create designable chemically active sites [61]. The 2-dimensional carbon material catalysts involved in this article are shown in Table S6.

Gr (Graphene)-based carbon-based catalysts are the most common 2-dimensional carbon materials. They rely on ultra-high specific surface area and rich defects and functional groups to form a “multiactive site, high conductivity” platform, which can coordinate the adsorption and dissociation of hydrogen molecules, sheet confinement, and electron/heat transport. At the same time, their continuous π conjugation gives them more advantages in low-temperature kinetics and cyclic stability. Verma et al. [62] investigated TiH_2_@Gr catalysts and reported that the synergistic interaction with MgH_2_ decreased the initial hydrogen desorption temperature to 204 °C and markedly reduced the activation barrier, effectively promoting hydrogen desorption kinetics. Subsequently, Song et al. [63] prepared FeOOH nanodots anchored onto graphene (FeOOH NDs@Gr), enabling the composite to store 6.0 wt% hydrogen at 200 °C under a pressure of 3.2 MPa within 60 min, maintaining 98.5% capacity retention after 20 cycles. To further enhance catalytic activity, Huang et al. [61] developed a Gn (graphene nanosheets)-supported bimetallic oxide catalyst Sc_2_O_3_/TiO_2_@Gn. When 10 wt% Sc_2_O_3_/TiO_2_@Gn was incorporated into MgH_2_, the composite showed markedly improved hydrogen absorption kinetics, absorbing 6.55 wt% hydrogen within 1 min at 300 °C and desorbing 5.71 wt% within 10 min. Moreover, the initial dehydrogenation temperature decreased to 140 °C. The composite exhibited superior cycling stability, maintaining 99.1% hydrogen storage capacity after 50 cycles. This enhanced performance was linked to the cooperative catalytic activity of Sc_2_O_3_ and TiO_2_, the presence of oxygen vacancies, and graphene’s role in preventing Mg particle agglomeration and catalyst dispersion. On this basis, Guemou et al. [64] designed a ternary Ni-Nb/rGO catalyst, demonstrating that doping MgH_2_ with 10 wt% Ni–Nb/rGO markedly decreased the activation energy to 57.8 kJ/mol. This composite absorbed 2.75 wt% hydrogen in 30 min at 75 °C under 3 MPa (Fig. 6E) and achieved 5.9 wt% hydrogen desorption in 10 min at 260 °C, with a maximum desorption of 6.41 wt% within just 5 min at 300 °C (Fig. 6F). This performance confirmed excellent hydrogen storage properties at moderate temperatures. Mechanistic studies indicated uniform dispersion of Ni/NiO/Nb_2_O_5_ nanoparticles on rGO sheets formed through ball milling, which improved dispersion and prevented agglomeration. During cycling, the Ni and Nb-based phases promoted hydrogen desorption, while rGO enhanced conductivity and stabilized catalyst dispersion (Fig. 6D).

MXenes are a class of 2-dimensional transition metal carbide/nitride/carbonitride flakes derived from a MAX phase by selectively removing the A layer. Leveraging a transition metal-containing layered carbon/nitride framework and programmable surface terminations (–O, –OH, –F), MXenes offer advantages in interfacial electronic structure control and active site construction. Li et al. [65] studied Ti_2_C MXene, demonstrating that multivalent Ti atoms promoted electron transfer processes between H^−^ and Mg^2+^, reducing the activation energy for hydrogen desorption from 107.3 to 71.7 kJ/mol, and lowering the onset temperature by 37 °C (from 327 to 290 °C). Liu et al. [66] developed a dual-MXene catalytic system of V_2_C and Ti_3_C_2_, which reduced the initial dehydrogenation temperature of MgH_2_ to 180 °C, achieving 5.1 wt% hydrogen desorption within 60 min at 225 °C, revealing synergistic interactions between the 2 MXene phases. Lu et al. [67] explored Nb_2_CT*_x_* MXene codoped with N/S elements, effectively lowering the hydrogen diffusion barrier, enabling hydrogen desorption of 4.78 wt% within 30 min at 225 °C, with overall enhanced storage performance. In recent research, Zhu et al. [68] fabricated an In@Ti-MX nanocomposite, composed of ultradispersed indium nanoparticles anchored onto single/few-layered Ti_3_C_2_ MXene nanosheets. By incorporating 15 wt% In@Ti-MX into MgH_2_, the composite absorbed 4.9 wt% hydrogen at 200 °C and desorbed 5.25 wt% hydrogen within 10 min at 325 °C, with reduced hydrogen desorption activation energy of 95.2 kJ/mol. Furthermore, the reaction enthalpy decreased to −66.2 kJ/mol, substantially improving thermodynamics. This performance arises from synergy between Ti_3_C_2_ MXene sheets and In/TiH_2_ phases, which catalyze H_2_ dissociation/recombination and provide diffusion pathways, thereby weakening Mg–H bonds. The disadvantages of using MXene as a carbon-based support are also notable. MXene requires the preparation of a MAX precursor, followed by etching, exfoliation, end group and oxidation control, and repeated cleaning and dispersion steps. This requires higher yield and stability management. While the cost of MXene is decreasing with the advancement of HF-free and continuous processes, it currently remains substantially higher than mainstream graphene.

Using MOF as a precursor for pyrolysis, flaky porous carbon nanosheets can be obtained in a directed manner. The thickness, pore size/specific surface area, heteroatom doping, and in situ anchoring of metal nanoparticles (or their oxides/carbides) can be designed by selecting layered/flaky MOFs, adjusting nitrogen-containing ligands and metal nodes, and controlling temperature and atmosphere. Lan et al. [69] prepared MOF-derived CN (carbon nitride) nanosheets. These nanosheets were codoped with disc-shaped Ni and TiO_2_ nanoparticles, and the composite was denoted as (Ni, TiO_2_)CN. Doping 10 wt% (Ni, TiO_2_)CN into MgH_2_ enabled rapid desorption, reaching 5.94 wt% H_2_ within 5 min at 300 °C. This also lowered the onset of dehydrogenation to 204 °C and the activation energy from 152.1 to 83.1 kJ mol^−1^. This also facilitated rapid hydrogen absorption, reaching 6.17 wt% within 10 min at 125 °C. Furthermore, the composite exhibited excellent cycling stability, retaining approximately 6.40 wt% H_2_ after 100 cycles. The performance improvement is attributed to the in situ formed Mg_2_Ni/Mg_2_NiH_4_ (acting as a “hydrogen pump”), redox-active Ti species that promote charge transfer, and N-doped carbon nanosheets that suppress particle agglomeration and disperse the active material.

### Three-dimensional carbon materials

Three-dimensional carbon materials focus on “bulk confinement and macroscopic formation”. Their hierarchical pores offer a large interfacial area and ample channels for hydrogen storage and desorption. They also maintain interparticle connectivity and efficient gas diffusion. The 3-dimensional carbon material catalysts involved in this article are shown in Table S7.

Carbon spheres are one of the most common 3-dimensional carbon materials. They can evenly and firmly anchor nanocatalytic particles on their surfaces and within their pores, providing pathways for rapid electron and heat transfer. Their spherical structure also acts as a buffer and “solid lubricant” during circulation. Their mature synthesis route, low equipment requirements, and undemanding raw material purity make them relatively scalable. Wang et al. [70] explored Ni@NCS (Ni-coated nitrogen-doped carbon spheres), which exhibited a hydrogen absorption capacity of 4.2 wt% at 100 °C within 60 min. Notably, the onset desorption temperature dropped to 246 °C—substantially lower than that of pure MgH_2_. Such improvement was attributed to reversible Mg_2_NiH_4_/Mg_2_Ni transformation (hydrogen pump effect), nitrogen-doping-induced active sites, and enhanced electron transfer, forming a triple-synergistic mechanism for improved hydrogen kinetics and cycling. Then, Wang et al. [71] prepared Ni@PHCNSs (porous hollow carbon nanospheres) loaded with uniformly dispersed Ni nanoparticles. This MgH_2_ composite exhibited a lowered initial hydrogen desorption temperature of 190 °C and maintained 94.1% hydrogen capacity after 50 cycles, confirming excellent cycling durability. Similarly, Soni et al. [72] studied Fe-HCS (Fe-loaded hollow carbon spheres), achieving a reduced desorption onset temperature of 225.9 °C and lowering activation energies for hydrogen absorption and desorption to 84.9 kJ/mol. The material also displayed outstanding stability over 24 cycles.

Other 3-dimensional carbon materials, including CMK-3 (mesoporous carbon), amorphous carbon, and graphene nanorods, have also exhibited promising catalytic activities. Cheng et al. [73] studied Pd_30_Ni_70_ supported on CMK-3, observing reduced hydrogenation and dehydrogenation activation energies of 65.9 and 78.9 kJ/mol, respectively. This composite achieved 4.0 wt% hydrogen absorption in 18,000 s at 100 °C, indicating notable low-temperature hydrogen storage performance. Yu et al. [74] found that amorphous carbon formed in situ reduced activation energy for hydrogen desorption to 87.2 kJ/mol, allowing MgH_2_ to absorb 5.62 wt% hydrogen within 3,600 s at 150 °C. Zhou et al. [75] developed an Fe–Ni alloy-decorated 3D graphene (Fe–Ni@3DG) catalyst. The resulting MgH_2_ composite exhibited hydrogen absorption of 6.35 wt% in just 100 s, and hydrogen desorption of 5.13 wt% in 500 s at 300 °C under 50 atm hydrogen pressure, maintaining excellent reversibility after 7 cycles. Notably, the ball-milled 3-dimensional graphene structure developed cracks and pores, markedly enhancing hydrogen diffusion pathways and interfacial catalytic activity. However, the cost of these carbon materials is generally high, limiting their widespread application.

The targeted design of 3-dimensional carbon-supported catalysts with multiple catalytic functions through the adjustable nanostructure of MOF materials is currently a popular research direction. Hu et al. [60] investigated Fe/C and Ni/C catalysts derived from MOFs. The MgH_2_–Fe/Ni/C composite showed reduced initial desorption temperature at 194 °C and hydrogen absorption of 6.2 wt% within 60 s at 150 °C. Similarly, Li et al. [76] fabricated flower-like CoFe@C catalysts via thermal decomposition of CoFe-based MOF precursors under nitrogen (Fig. 6G). These catalysts demonstrated excellent catalytic efficiency for MgH_2_-based hydrogen storage. The composite exhibited rapid hydrogen absorption, achieving a capacity above 6 wt% within 2,000 s at 200 °C and 3 MPa (Fig. 6H). Additionally, it desorbed 6.0 wt% hydrogen within 400 s at 300 °C, markedly outperforming pristine MgH_2_ (Fig. 6I). Notably, the CoFe@C catalyst lowered the activation energy for hydrogen desorption by 38%, decreased the onset desorption temperature to 175.9 °C, and retained reversible storage capacity of 6.1 wt% after 20 cycles, indicating superior cycle stability. Mechanistic analysis confirmed that improved catalytic performance resulted from synergistic interactions among α-Fe, Mg_2_CoH_5_, and Mg_2_Co phases generated in situ, in combination with conductive carbon matrices that facilitated hydrogen dissociation and diffusion without compromising catalyst integrity (Fig. 6G). Guo et al. [77] further demonstrated that Ni–MOF-derived carbon-supported Mg–Mg_2_Ni composites decreased activation energy to 77.6 kJ/mol, retaining 6.0 wt% hydrogen after 10 cycles, validating the effectiveness of MOF-derived carbon nanostructures in enhancing hydrogen storage kinetics of MgH_2_.

## Other Compound Catalysts

Catalysts such as metal nitrides, carbides, borides, and MOFs have shown remarkable capability in promoting MgH_2_-based hydrogen storage performance. These materials effectively lower hydrogen desorption temperatures, enhance hydrogen diffusion kinetics, and improve cycle durability. Other types of catalysts involved in this article are shown in Table S8.

**Fig. 7.**
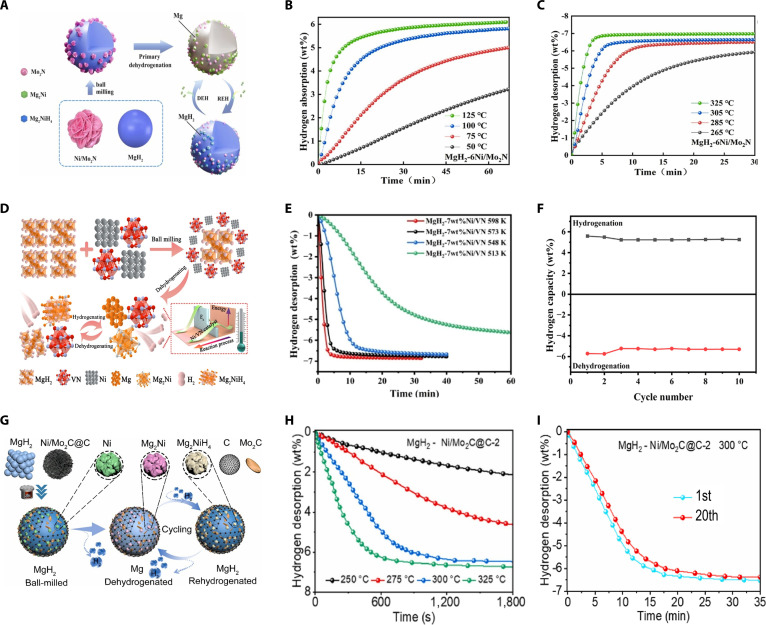
(A) Schematics of the hydrogenation and dehydrogenation mechanisms of MgH_2_ doped with Ni/Mo_2_N. (B) Hydrogenation profiles and (C) corresponding dehydrogenation profiles of MgH_2_ doped with Ni/Mo_2_N catalyst at different temperatures [78]. (D) Schematics of the hydrogenation and dehydrogenation mechanisms of MgH_2_ doped with Ni/VN. (E) Dehydrogenation behavior of MgH_2_ doped with 7 wt% of Ni/VN at different temperatures. (F) Cycling behavior of MgH_2_ with 7 wt% Ni/VN at 573 K [79]. (G) Schematics of the hydrogenation and dehydrogenation mechanisms of MgH_2_ doped with Ni/Mo_2_C@C-2. (H) Dehydrogenation behavior of MgH_2_ doped with 7.5 wt% Ni/Mo_2_C@C-2 at different temperatures. (I) Cycling behavior of MgH_2_ with 7.5 wt% Ni/Mo_2_C@C-2 at 300 °C [81].

Metal nitrides combine high activity and durability. The strong hybridization of lattice nitrogen with transition metals shifts the metal’s d-band downward and narrows it, placing hydrogen adsorption within the Sabatier optimal range. Simultaneously, the metal (Lewis acid) and adjacent nitrogen (Lewis base) sites heterolytically split H_2_ into M–H and N–H. The resulting hydrogen spillover rapidly transports H and markedly lowers the barrier for Mg–H bond cleavage and recombination. Sun et al. [78] synthesized Ni/Mo_2_N catalysts via a hydrothermal approach. After doping into MgH_2_, the initial dehydrogenation temperature dropped to 186.3 °C, about 135.8 °C below pure MgH_2_. The catalyst markedly enhanced hydrogen storage kinetics, allowing the desorption of 6.80 wt% hydrogen within 5 min at 325 °C and 6.09 wt% within 10 min at 285 °C (Fig. 7C). Additionally, the composite absorbed 5.46 wt% hydrogen in 15 min at 125 °C, demonstrating substantial improvement over pristine MgH_2_ (Fig. 7B). Structural characterization confirmed that Ni/Mo_2_N promoted reversible Mg_2_Ni/Mg_2_NiH_4_ formation and introduced stable active sites for rapid hydrogen diffusion pathways (Fig. 7A). Wu et al. [79] synthesized Ni/VN catalysts through solvothermal techniques. MgH_2_ doped with 7 wt% Ni/VN exhibited a lower onset hydrogen desorption temperature of 205 °C, approximately 168 °C lower compared with pure MgH_2_ (373 °C), releasing 6.4 wt% hydrogen within 3 min at 325 °C and 5.6 wt% within 1 h at 240 °C (Fig. 7E). It also absorbed 6.0 wt% hydrogen within 5 min at 150 °C, maintaining a capacity retention of 91.6% after 10 cycles at 300 °C (Fig. 7F). XRD (x-ray diffraction) analyses confirmed the generation of Mg_2_Ni/Mg_2_NiH_4_ phases, and XPS (x-ray photoelectron spectroscopy) revealed uniformly dispersed Ni nanoparticles. The VN component provided stable active sites and enhanced hydrogen diffusion, synergistically improving the hydrogen storage efficiency (Fig. 7D).

Metal carbides similarly utilize p–d hybridization with nonmetallic (C) atoms to shift the metal’s d-band downward, placing hydrogen adsorption within the Sabatier optimum. This heterolytic H_2_ synergistically splits H_2_ between metal and nonmetallic sites, promoting hydrogen diffusion. However, nitrogen’s higher electronegativity enhances hydrogen pumping capacity, while carbon exhibits stronger covalent interactions with metals, resulting in improved electron transport and sintering resistance. Yao et al. [80] investigated the synergistic role of transition metal carbides (TiC, ZrC, and WC) combined with Mg_2_NiH_4_, identifying TiC as most effective. The MgH_2_–TiC composite showed a hydrogen desorption onset at 216 °C and retained 95% hydrogen storage capacity after 10 cycles. The cooperative effects between Mg_2_NiH_4_ and TMCs enhanced kinetics by facilitating electron transfer, markedly reducing activation energies. Yuan et al. [81] fabricated Ni/Mo_2_C@C catalysts by pyrolysis of polydopamine-coated NiMoO_4_ precursors. The resulting composite displayed an onset desorption temperature decreased by 83 °C, reduced activation energy to 97.22 kJ/mol, and desorbed 6.35 wt% hydrogen within 20 min at 300 °C (Fig. 7H). Moreover, the hydrogen absorption capacity was stable at 97.8% after 20 cycles (Fig. 7I). The catalyst enhanced hydrogen cycling through the generation of Mg_2_NiH_4_ phases, effectively weakening Mg–H bonds, while Mo_2_C provided stable active sites and promoted electron transfer, improving the kinetics of hydrogen desorption. Graphitized carbon further prevented particle aggregation and accelerated diffusion, markedly enhancing hydrogen storage performance (Fig. 7G).

MOF catalysts, relying on their ultra-large specific surface area, designable metal-ligand sites, and nano-confinement effects, can simultaneously achieve low-barrier H_2_ cracking and hydrogen overflow, enhance electron transfer, and inhibit sintering. Shao et al. [82] demonstrated that thermally stable Ni-BTC300 MOF catalysts decreased hydrogen desorption barriers, enabling MgH_2_ doped with 10 wt% Ni-BTC300 to desorb 5.14 wt% hydrogen in 3 min at 300 °C, markedly higher than pure MgH_2_ (0.09 wt%) under identical conditions. Li et al. [83] studied Ni-MOF@Pd catalysts, enhancing dehydrogenation through Mg_2_Ni/Mg_2_NiH_4_-mediated hydrogen pumping and formation of Mg–Pd alloys. The composite’s initial hydrogen desorption temperature was reduced to 181 °C. Lu et al. [84] reported that MOF–V catalysts reduced initial dehydrogenation temperature to 190.6 °C. During cycling, partial conversion to metallic vanadium enhanced interfacial catalysis and structural stability via nano-confinement effects.

## Machine Learning and First-Principles Approaches for MgH_2_ Hydrogen Storage

Current research on solid-state hydrogen storage catalysts rarely uses in situ techniques such as XRD or XAS (x-ray absorption spectroscopy) to explore phase transitions during cycling. This limits real-time insights into dynamic structures and hinders the rational design of more efficient catalyst systems. Recent advances in computation have placed density functional theory (DFT) and machine learning (ML) at the core of MgH_2_-based hydrogen storage research. Combining experimental characterization with DFT and ML can shorten the trial-and-error cycle, provide atomic-level explanations for rate-limiting steps, and enable data-driven screening of complex composition-structure spaces. ML expands the scope of exploration to thousands, while DFT quantifies the thermodynamics and kinetics of active sites, which are ultimately verified by experimental characterization. The most effective strategy is to couple them into a single workflow for hypothesis generation, validation, and inverse design [85–89].

**Fig. 8.**
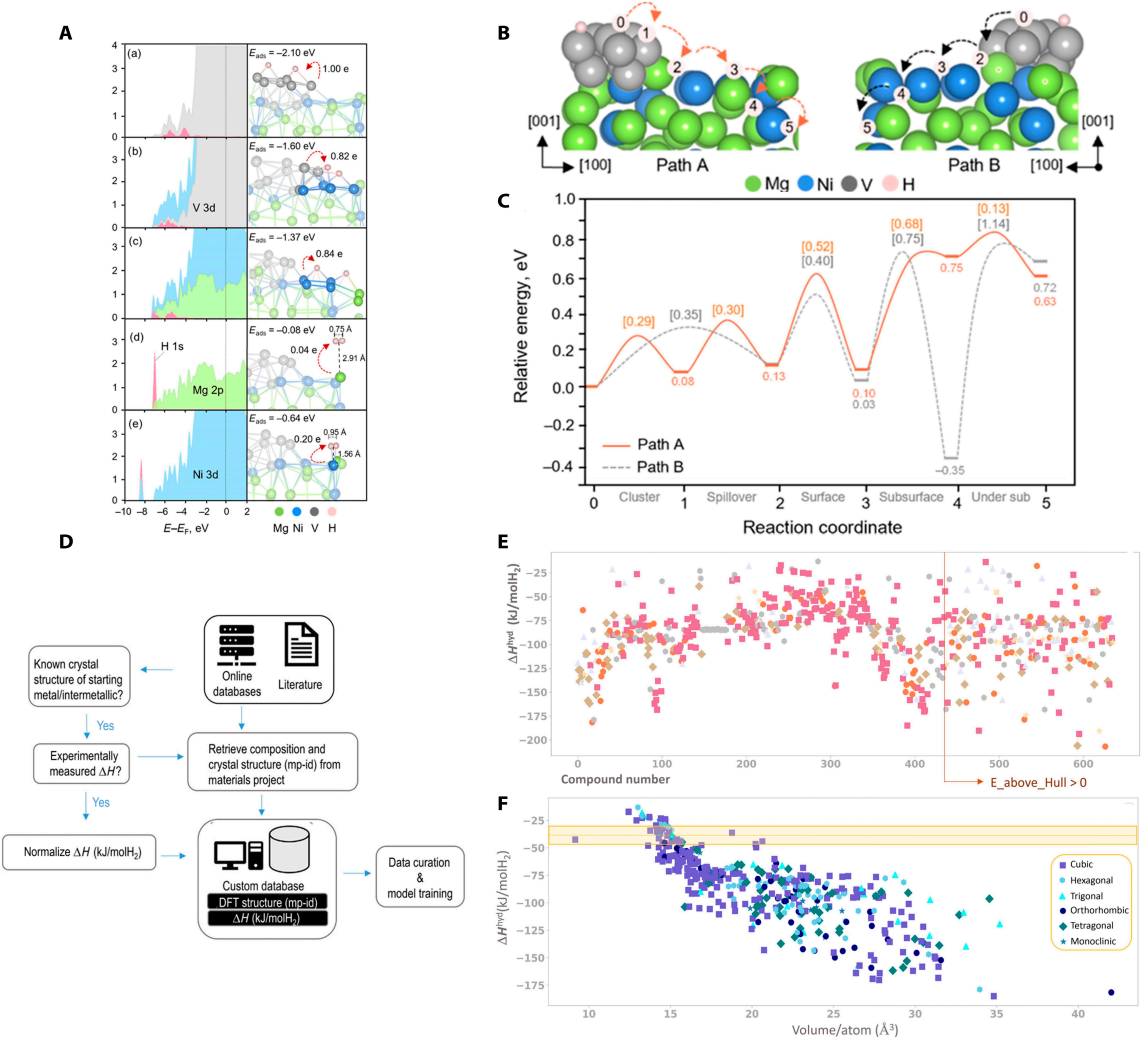
(A) Projected density of states (PDOS) and atomic configurations of hydrogen adsorption at the V cluster, interface, hollow site, atop Mg, and atop Ni on the V_10_/Mg_2_Ni surface. (B) Atomic models of hydrogen diffusion along Path A and Path B from the V cluster to the under-subsurface region. (C) Reaction energy profiles corresponding to Path A and Path B, with diffusion barriers and relative energies labeled [90]. Reproduced with permission [90]. Copyright 2025, American Chemical Society. (D) MetalHydrideEnth model building process. (E) Distribution of predicted hydride formation enthalpies for 636 Mg-containing intermetallic compounds screened via the model. (F) Correlation between hydride formation enthalpy and atomic volume, highlighting a structural descriptor range favorable for near-ambient hydrogen storage (shaded area) [93].

DFT studies have clarified key microscopic processes—including H_2_ adsorption/dissociation, H diffusion, and Mg–H bond modulation—under realistic catalytic motifs (doping, interfacial alloys, and reconstructed surfaces). For example, Khajondetchairit et al. [90] showed that Ni/V decoration on Mg_2_Ni surfaces enables barrierless H_2_ dissociation via strong 3d–1s hybridization (Fig. 8A). It also reduces the H-diffusion barrier from 0.49 to 0.13 eV through bridged active sites and surface reconstruction (Fig. 8B and C). Reddad et al. [91] further demonstrated element-specific effects: Cu substitution markedly lowers the dehydrogenation barrier and temperature, whereas Zn showed limited impact due to insufficient orbital overlap with Mg. In another study, Didi et al. [92] examined ternary hydrides MgV_3_H_8_ and MgFe_3_H_8_ via CI-NEB calculations, identifying favorable phonon stability and low H-diffusion barriers (0.14 eV), though dehydrogenation temperatures above 599 K still exceeded DOE targets. Collectively, these studies show that DFT can (a) identify and tune active sites, (b) compute reaction and transport barriers, and (c) rationalize electronic-structure descriptors correlated with hydrogen kinetics. However, most models remain idealized, limiting transferability to real systems with nanoparticle agglomeration, by-product accumulation, and multielement synergies. The high computational cost of exhaustive sampling also constrains throughput.

ML has recently been adopted to overcome this bottleneck by learning structure–property relationships from computational and experimental data. Batalović et al. [93] developed the graph neural network model MetalHydrideEnth to screen over 600 Mg-containing intermetallics (Fig. 8D), identifying MgBe_13_ as a lightweight and stable candidate (−42.5 kJ·mol^−1^ H_2_ enthalpy) (Fig. 8E), and revealing atomic packing density as a structural descriptor strongly correlated with formation enthalpy (Fig. 8F). In another contribution, Dong et al. [94] established a supervised ML framework based on a curated database of 826 Mg-alloy hydride samples, training regression models with maximum hydrogen absorption and desorption as outputs. The GBR (gradient boosting regression) and MLP (multilayer perceptron) models achieved high accuracy (*R*^2^ = 0.947 and 0.922). SHAP (Shapley Additive Explanations) analysis highlighted electronegativity mismatch, valence-electron concentration, and ball-milling parameters as critical descriptors for Mg-based hydrogen storage. The models successfully predicted promising candidates such as 96Mg–4Sm and 95Mg–1Ni–4Sm alloys, with hydrogen absorption and desorption capacities exceeding 6 wt% below 300 °C. Similarly, Jia et al. [95] used interpretable recurrent neural networks with SHAP analysis to identify milling time and processing temperature as key descriptors for Mg(BH_4_)_2_ composites, achieving dehydrogenation onset at 112.2 °C and 8.5 wt% capacity. These cases illustrate that ML can accelerate discovery, reveal hidden correlations, and guide synthesis. Yet, challenges persist: dataset scarcity, heterogeneous reporting standards, and limited physical interpretability often restrict generalization when ML is used alone.

The most promising path is the synergistic integration of DFT and ML. For instance, Li et al. [96] combined COHP (crystal orbital Hamiltonian population)-derived DFT descriptors with ML regressors, achieving <0.04 eV error in predicting dehydrogenation barriers of Ni/Mn-doped MgH_2_ and identifying optimized doping pathways with barriers as low as 1.81 eV. Jiang et al. [97] used DFT to design NLi_4_–B-doped graphene/MgH_2_ heterojunctions, then integrated ML to screen 132 Mg_1−*x*_Me*_x_*H_2_ alloys for tailored desorption temperatures. Validated candidates included Mg_0.875_Li_0.125_H_2_ and Mg_0.875_Be_0.125_H_2_ with 400 to 500 K desorption ranges. In another effort, Lee et al. [98] used a GAN (Generative Adversarial Network) to inverse-design 2D MgH_2_ sheets; subsequent DFT uncovered a previously unknown P−4m2 phase. With Li decoration, it achieved 6 wt% gravimetric capacity and an adsorption energy of −0.105 eV. These hybrid approaches demonstrate how ML can explore broad compositional and structural spaces, while DFT validates physical mechanisms, enabling inverse design of Mg-based catalysts and heterostructures.

Looking forward, several directions deserve emphasis. First, unified databases integrating DFT outputs (energies, phonons, and diffusion paths) with standardized experimental data (activation energies, cycling metrics, and failure modes) are urgently needed. Second, physically informed ML models that encode symmetry, thermodynamic limits, and defect chemistry will enhance extrapolation reliability. Third, multiscale simulations bridging atomistic kinetics with mesoscale transport (grain growth and MgO/MgS by-products) and reactor-level performance will connect computational screening with real applications. Finally, applying integrated DFT–ML strategies to multicomponent and high-entropy catalysts in nano-confined carbon/oxide frameworks offers a pathway to cost-effective, durable MgH_2_ systems that align with DOE targets. Overall, the convergence of DFT and ML should be considered not as auxiliary tools but as the central design engine for next-generation MgH_2_ hydrogen storage catalysts.

## Discussion

**Fig. 9.**
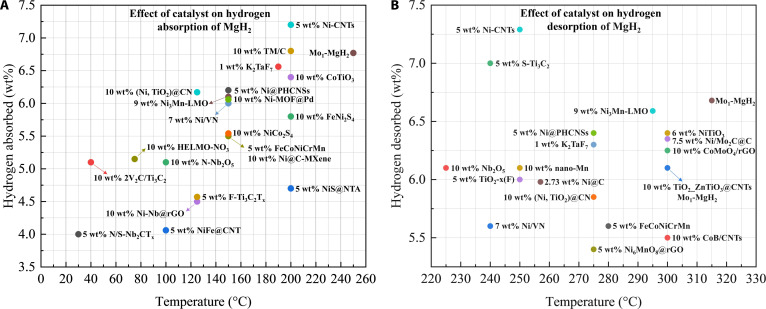
The highly efficient catalysts’ (A) hydrogen absorption and (B) hydrogen desorption.

Figure 9 compiles several high-performance catalysts in this review. These systems typically couple rapid H_2_ dissociation with engineered Mg/MgH_2_ interfaces, which shorten diffusion distances and preserve stability under repeated cycling.

High-entropy materials leverage dense local active sites, distortion-induced fast-diffusion channels, and the sintering resistance afforded by configurational entropy. As a result, they lower the barriers for both H_2_ activation and Mg–H bond breaking/forming while sustaining interfacial activity. For example, with HELMO-NO_3_ catalysis, MgH_2_ absorbs 6.68 wt% hydrogen absorption within 3 min at 315 °C and 5.15 wt% at 75 °C. The MgH_2_–FeCoNiCrMn alloy absorbs 5.5 wt% in 0.5 min at 150 °C and desorbs 5.8 wt% in 4 min at 280 °C, with 99% capacity retention after 50 cycles. Nonetheless, achieving and stabilizing near-atomic, single-phase solid solutions usually requires high-purity feedstocks and energy-intensive processing. These constraints raise manufacturing costs and narrow the practical window for scale-up.

Metal fluorides pair strong Lewis acidity with the in situ formation of fluorinated active surfaces. With K_2_NbF_7_, the MgH_2_ composite shows a desorption activation energy of 96.3 kJ mol^−1^, desorbs 5.2 wt% H_2_ in 5.6 min at 320 °C, and still absorbs 4.7 wt% at 150 °C within 30 min. However, their preparation and use require strictly anhydrous handling and corrosion-resistant equipment. In humid environments, moisture-induced hydrolysis can generate HF, which shortens catalyst lifetime and adds nontrivial safety and environmental protection costs.

Single-atom and defect-engineered catalysts further accelerate kinetics: With Ni@TiO_2_ catalysis, MgH_2_ achieves 6.53 wt% absorption in 10 s and retains 97% capacity after 100 cycles. The activation energies drop to 64 kJ mol^−1^ (desorption) and 35 kJ mol^−1^ (absorption). Nevertheless, robust anchoring of isolated metal atoms at defect or coordination sites demands stringent control over precursors, atmosphere, and temperature. Such controls increase processing complexity and cost. Maintaining comparable defect densities and uniform single-atom dispersion at larger batch sizes remains challenging, and this currently limits reproducible manufacturing.

Carbon-based composites strike a favorable balance between activity and durability. The MgH_2_–Sc_2_O_3_/TiO_2_@graphene composite exhibits a dehydrogenation onset to 140 °C and maintains 99% capacity after 50 cycles. The MgH_2_ composite catalyzed by ternary Ni–Nb/rGO exhibits a low apparent activation energy (57.8 kJ mol^−1^), low-temperature absorption (2.75 wt% at 75 °C, 3 MPa, 30 min), and rapid desorption at moderate temperatures—benefits that derive from oxygen-vacancy chemistry, multiphase synergy, and rGO-assisted dispersion and transport. However, incorporating carbon-based composites at high loadings substantially dilutes the usable hydrogen-storage capacity. In addition, validation under high-density compaction conditions representative of on-board applications remains limited. The effects of the carbon framework on mass transport, heat conduction, and hydrogen absorption/desorption behavior under high pressure have not been systematically elucidated.

Collectively, these catalysts accelerate H_2_ activation via defect-rich interfaces, Lewis-acid fluorides, single-atom sites, and conductive carbon scaffolds. However, challenges remain in scalable synthesis, cost, handling safety, and maintaining activity under practical conditions—issues that must be addressed for widespread application in hydrogen storage systems.

## Conclusion

MgH_2_ offers a gravimetric capacity of 7.6 wt% and is therefore an attractive solid hydrogen carrier. Its practical use, however, is constrained by high thermodynamic stability. Doped catalysts can reroute the reaction pathway and lower activation barriers. Through interfacial charge transfer, they weaken the Mg–H bond, thereby promoting H_2_ dissociation, spillover, and atomic diffusion. During cycling, new interfacial phases often emerge—such as Mg_2_Ni/Mg_2_NiH_4_, MgF_2_, or MgS—producing multiphase architectures that suppress grain growth and preserve active surface area.

In recent years, several families of efficient catalysts have emerged to tackle the (de)hydrogenation-kinetics bottleneck of MgH_2_. They promote interfacial charge transfer and band-structure modulation. They introduce defects and well-defined active sites. They also exploit nanoscale confinement. Representative systems include high-entropy catalysts, SACs, and carbon-based composite supports.

1. High-entropy catalysts utilize lattice distortion and multielement synergistic effects to promote interfacial electron transfer and weaken Mg–H bonds. The multielement composition provides abundant active sites and enhances both hydrogen absorption and desorption kinetics. Moreover, the intrinsic compositional disorder helps inhibit particle growth and maintains catalyst stability during cycling.
2. SACs operate via the “single atom–defect–support” interplay. Isolated metal atoms anchored on defects serve as highly active centers for hydrogen dissociation. The unique coordination environment and electronic interactions facilitate efficient charge transfer, sharply reducing activation energies without compromising the system’s thermodynamic stability. Additionally, these catalysts offer tunable selectivity and enhanced durability by minimizing atom migration and aggregation.
3. Carbon-based matrices provide high surface area, electrical conductivity, and excellent dispersibility. The robust structural framework can host transition metal nanoparticles or single atoms, stabilizing them against sintering and aggregation. The conductive carbon matrix promotes fast electron transfer and can synergistically regulate hydrogen absorption and desorption kinetics.
4. Other types of catalysts discussed in this article, such as elemental metals, metal halides, metal sulfides, and metal carbides, have certain advantages in terms of material sources and preparation methods, and may play a role in the initial large-scale hydrogen storage applications. However, their catalytic effects are relatively limited, and we look forward to further improvements in catalytic effects and cycle performance in the future.

Looking ahead, multielement high-entropy catalysts, SACs, and carbon-supported nanoscale confinement strategies are likely to be the mainstream research areas. Moreover, integrated, data-driven design paradigms will be crucial:

1. Co-ball milling MgH_2_ with a high-entropy precursor to construct a multiphase, defect-rich contact interface in situ. During cycling, multicomponent species with variable valence states can be partially reduced, serving as both active sites and electron and hydrogen transport channels.
2. Anchoring transition metal single atoms onto a robust support (e.g., TiO_2_ or fluoride nanosheets) achieves atomic-level dispersion and strong interfacial coupling, thereby promoting the H_2_ cracking-escape-diffusion process and inhibiting grain coarsening.
3. Utilizing the dispersibility and stability of carbon materials to load nanoscale transition metal or solid solution nanoparticles, while simultaneously modulating thermodynamics and improving kinetics, enhances their cycling stability.
4. Combining in situ characterization, DFT, and ML can reveal dynamic structural evolution processes, predict reaction energies, and identify promising components before synthesis, thereby substantially reducing experimental costs and accelerating catalyst discovery and optimization.
