# Supplementary material for: Advances in Catalysts for Magnesium-Based Hydrogen Storage Materials
Source: Research (Wash D C). 2025 Dec 22;8:1036. doi: 10.34133/research.1036 (PMC12719564; doi:10.34133/research.1036)
Supplement: Supplementary 1 — Tables S1 to S8 [file research.1036.f1.docx]

**Advances in catalysts for magnesium-based hydrogen storage materials**

Yong Zhu^1, 2, 3#^, Wenhao Ma^2#^, Xingzai Chai^2^, Yunpeng Gu^2^, Wenqi Wu^2^, Haisheng Chen^2, 3, 4*^, Ting Zhang^2, 3, 4,5*^

^1^ School of Energy and Mechanical Engineering, Nanjing Normal University, Nanjing 210042, China.

^2^ Nanjing Institute of Future Energy System, Nanjing 211135, China.

^3^ University of Chinese Academy of Sciences, Nanjing 211135, China.

^4^ University of Chinese Academy of Sciences, Beijing 100049, China.

^5^ Institute of Engineering Thermophysics, Chinese Academy of Sciences, Beijing 100190, China.

* Address correspondence to: [zhangting@iet.cn](mailto:zhangting@iet.cn) (T.Zhang), [chen_hs@iet.cn](mailto:chen_hs@iet.cn) (H.C.)

^#^ These authors contributed equally to this work.

**Table 1** Improving hydrogen storage in MgH_2_ using metal and alloy catalysts

| **Composite Materials** | **Preparation method** | **Hydrogen adsorption** | **Hydrogen desorption** | **Remaining capacity/ cycles** | **Deh E_a_**  **(kJ/mol)** | **Ref.** |
| --- | --- | --- | --- | --- | --- | --- |
| 10 wt% nano-Mn | Chemical reduction | - 2.0 wt% at 50°C, 30 min. - 6.0 wt% at 300°C. | - 6.1 wt% at 250°C, 25 min. - Onset desorption at 175°C. | 92%/20 | / |  |
| Mo_1_-MgH_2_ | Impregnation  ball milling | - 6.77 wt% at 250°C, 1 min. | - 5.85 wt% at 250°C, 4 h. - Onset desorption at 218°C. | ≈100%/10 | 110 |  |
| nano-FeCo | Wet-chemical  ball milling | - 6.7 wt% at 300°C, 1 min. | - 6.0 wt% at 300°C, 9.5 min. | 91.4%/10 | 65.3 |  |
| 5 wt% FeCoNiCrMn | ball milling | - 5.5 wt% at 150°C, 0.5 min. | - 5.6 wt% at 280°C, 10 min. | 98.6%/50 | 90.2 |  |
| 10 wt% FeCoNiLa | electrodeposition  ball milling | - >6.5 wt% at 325°C, 5 min | - Onset desorption at 281.6°C. | 98.2%/20 | 103.92 |  |
| Ni-N-C | Quasi-solid-state  template method | - No mention. | - 5.4 wt% at 300°C, 5 min. | 97%/10 | 87.2 |  |
| Ti | ball milling | - 2.7 wt% at 300°C, 10 bar, 7 min. | - 2.7 wt% at 350°C, 50 mbar, 6 min. | / | / |  |
| 2 mol% Ni (nano) | ball milling | - 5.3 wt% at 300°C, 10 bar, 5 min. | - 5.3 wt% at 300°C, 50 mbar, 4 min. | / | / |  |

**Table 2** Improving hydrogen storage in MgH_2_ using metal oxide catalysts

| **Composite Materials** | **Preparation method** | **Hydrogen adsorption** | **Hydrogen desorption** | **Remaining capacity/ cycles** | **Deh E_a_**  **(kJ/mol)** | **Ref.** |
| --- | --- | --- | --- | --- | --- | --- |
| 5 wt% TiO_2_ (NR750) | Alkaline hydrothermal | - 3.9 wt% at 350°C, 10 bar, 5 min. | - 3.9 wt%, 0.1 bar, 3 min. | / | / |  |
| 5 wt% TiO_2_ (TF70) | Hydrothermal method | - 5.3 wt% at 200°C, 44 s. | - 6.4 wt% at 300°C, 700 s. | 97.6%/10 | 76.1 |  |
| 15 wt% Ni_0.034_@TiO_2_ | Molten salt method | - 6.53 wt% at 300°C, 10 s. | - 4.6 wt% at 300°C, 5 min. | 97.26%/100 | 64.35 |  |
| 10 wt% N-Nb_2_O_5_ | Solvothermal method | - 5.1 wt% at 100°C, 50 atm, 3 min. | - 6.3 wt% starting at 170°C. - Terminating at 268°C. | / | 98 |  |
| 10 wt% NiV_2_O_6_ | Hydrothermal method | - 5.59 wt% at 150°C, 50 min. | - 5.2 wt% at 300°C, 10 min. - Onset desorption at 227°C. | 90%/50 | 92.9 |  |
| 10 wt% CoMoO_4_/rGO | Hydrothermal method | - 4.2 wt% at 150°C, 3 MPa, 20 min. - Begins absorption below 80°C. | - 6.25 wt% at 300°C, 10 min. - Onset dehydrogenation at 204°C. | ≈98%/10 | 123.46 |  |
| 9 wt% Ni_3_Mn-LMO | Hydrothermal  ball milling | - 6.10 wt% at 150°C, 600 s | - 4.44 wt% at 235°C in 60 min. - Onset dehydrogenation at 190°C. | 92.8%/20 | 86.88 |  |
| 10 wt% HELMO-NO_3_ | Ball milling | - 5.15 wt% at 75°C in 50 min. | - Onset dehydrogenation at 184.1°C. | 97.8%/50 | 54.59 |  |

**Table 3** Improving hydrogen storage in MgH_2_ using metal halide catalysts

| **Composite Materials** | **Preparation method** | **Hydrogen adsorption** | **Hydrogen desorption** | **Remaining capacity/ cycles** | **Deh E_a_**  **(kJ/mol)** | **Ref.** |
| --- | --- | --- | --- | --- | --- | --- |
| 15 wt% HfCl_4_ | Ball milling | - 5.5 wt% at 300°C, 5 min. | - 4.5 wt% at 300°C, 5 min. - Onset desorption at ~ 265°C. | / | 102.0 |  |
| 15 wt% VCl_3_ | Cryogenic ball milling | - 5.6 wt% at 300°C, 1 h. | - 6.0 wt% at 350°C, 20 min. - Onset desorption at ~ 280°C. | / | 123.11 |  |
| 10 wt% LaCl_3_ | Ball milling | - 5.1 wt% at 300°C, 2 min. | - 4.2 wt% at 320°C, 5 min. - Onset desorption at 300°C. | / | 143.0 |  |
| 10 wt% CeCl_3_ | Ball milling | - 5.2 wt% at 280°C, 5 min. | - 5.5 wt% at 350°C, 10 min. - Onset desorption at 300°C. | / | 149.0 |  |
| 5 wt% K_2_NbF_7_ | Ball milling | - 4.7 wt% at 150°C, 30 min. - 5.1 wt% at 320°C, 43 s. | - 5.2 wt% at 320°C, 5.6 min. - Onset desorption at 255°C. | 99.7%/5 | 96.3 |  |
| 1 wt% K_2_TaF_7_ | Ball milling | - 6.56 wt% at 190°C. - 7.42 wt% at 250°C. | - 6.3 wt% at 275°C, 1000 s. - Onset desorption at 234.3°C. | 96.7%/10 | 107.2 |  |
| 10 wt% BaCoF_4_ | Ball milling | - 6.4 wt% at 300°C, 5 min. | - 5.0 wt% at 300°C, 30 min. - Onset desorption at 265°C. | / | 100.0 |  |
| NbF_5_ + pBN | Calcination  Melt impregnation | - No mention. | - 6.5 wt% at 300°C, 10 min. - Onset desorption at 230°C. | / | / |  |
| 5 wt% TiO_2-x_(F) | Solvothermal  Hydrogen reduction | - 3.0 wt% at 50°C, 120 min. - 6.0 wt% at 150°C, 1 min. | - 6.0 wt% at 250°C, 6 min. - Onset desorption at 189°C. | / | 119.96 |  |
| 10 wt % YF_3_ + Ni/C | Ball milling | - 5.18 wt% at 50°C, 4 h. | - 5.74 wt% at 250°C, 30 min. | 98.4%/30 | 74.56 |  |

**Table 4** Improving hydrogen storage in MgH_2_ using metal sulfide catalysts

| **Composite Materials** | **Preparation method** | **Hydrogen adsorption** | **Hydrogen desorption** | **Remaining capacity/ cycles** | **Deh E_a_**  **(kJ/mol)** | **Ref.** |
| --- | --- | --- | --- | --- | --- | --- |
| 5 wt% TiS_2_ | Commercial | - 5.3 wt% at 100°C, 5 min. | - 5.9 wt% at 300°C, 10 min. | ≈100%/20 | 50.8 |  |
| 16.7 wt% Fe_7_S_8_ | Hydrothermal method | - 4.0 wt% at 200°C, 30 min. | - 4.4 wt% at 350°C, 30 min. - Onset desorption at 147°C. | / | 130.0 |  |
| 5 wt% NiS@NTA | Hydrothermal, Carbonization | - 4.7 wt% at 200°C, 100 s. - 6.0 wt% at 400°C, 100 s. | - 3.5 wt% at 300°C, 60 min. - Onset desorption at 235°C. | 96.4%/20 | 67.3 |  |
| Ni@TiS_2_ | Solution method | - 4.60 wt% at 300°C, 60 s. | - 4.60 wt% at 300°C, 240 s | 98%/50 | 79.4 |  |
| 10 wt% NiCo_2_S_4_ | Hydrothermal method | - 5.54 wt% at 150°C, 1 min. | - 6.11 wt% at 325°C, 5 min. - Onset desorption at 203°C. | 98.8%/10 | 86.50 |  |
| 10 wt% FeNi_2_S_4_ | Solvothermal method | - 4.02 wt% at 100°C, 1 h. - 5.8 wt% at 200°C, 10 s. | - 1.92 wt% at 300°C. - Onset desorption at 267°C. | ≈100%/10 | 65.5 |  |
| 10 wt% FeCoS@C | MOF-derived method | - 6.78 wt% at 300°C, 60 s. - 4.56 wt% at 200°C, 900 s. | - Onset desorption temperature reduced from 347°C to 277°C. | / | 91.9 |  |
| 10 wt% FeNi_3_-S | MOF-derived method | - 2.49 wt% at 100°C, 30 min. | - 6.57 wt% at 325°C, 1000 s. - Onset desorption at 202°C. | 94.2%/20 | 98.6 |  |
| 5 wt% S-Ti_3_C_2_ | Heat treatment  in Ar/H_2_S | - 6.8 wt% at 200°C, 25 min. - 5.1 wt% at 200°C, 68 s. | - 7.0 wt% at 240°C, 7 min. - Onset desorption at 173°C. | 95.6%/10 | 92.53 |  |

**Table 5** Improving hydrogen storage in MgH_2_ using 1D carbon-based composite catalysts

| **Composite Materials** | **Preparation method** | **Hydrogen adsorption** | **Hydrogen desorption** | **Remaining capacity/ cycles** | **Deh E_a_**  **(kJ/mol)** | **Ref.** |
| --- | --- | --- | --- | --- | --- | --- |
| 76.8 wt% MgH_2_@BCNTs | Impregnation  Hydrogenation | - 5.79 wt% at 250°C, 5 min. | - 5.70 wt% at 275°C, 1 h. - 5.79 wt% at 300°C, < 30 min. | 99.8%/10 | 97.97 |  |
| 5 wt% Ni-CNTs | Hydrothermal  Calcination | - 7.2 wt% at 200°C, 30 min. | - 7.29 wt% at 250°C, 15 min. - Onset desorption at 200°C. | ≈100%/10 | 74.8 |  |
| 10 wt% TiO_2__ZnTiO_3_@CNTs | Hydrothermal Calcination | - 4.47 wt% at 125°C, 60 min. - Onset absorption at < 50°C. | - 6.1 wt% at 300°C, 10 min. - Onset desorption at 197°C. | 97.0%/20 | 108.33 |  |
| 5 wt% NiFe@CNT | Calcination | - 4.06 wt% at 100°C, 30 min. - 3.25 wt% at 75°C, 30 min. | - Onset desorption at 225°C. | / | 49.7 |  |
| 6 wt% VTiFe + 3 wt% CNTs | Mechanical alloying  Two-step ball milling | - 5.9 wt% at 175°C, 30 min. - 2.3 wt% at 75°C, 30 min. | - 5.8 wt% at 250°C. - Onset desorption at 179°C. | 97.3%/31 | 69.8 |  |
| 1 wt% Co@Pd-CNTs | Hydrothermal  Calcination | - 7.30 wt% at 200°C, 8 h. - 7.29 wt% at 250°C, 15 min. | - 7.30 wt% at 250 °C, 30 min. - Onset desorption at 200°C. | ≈100%/10 | 81.93 |  |
| 10 wt% Ni@pCNF | MOF-derived carbonization | - 2.2 wt% at 100°C, 120 min. - 4.3 wt% at 250°C, 10 min. | - 3.0 wt% at 250°C, 120 min. - Onset desorption at 200°C. | 95.4%/10 | 96.58 |  |
| 10 wt% Fe + Ni/C | MOF-derived carbonization | - 6.8 wt% at 200°C, 60 s. - 5.7 wt% at 100°C, 1 h. | - 6.5 wt% at 323°C. - Onset desorption at 194°C. | 99.8%/10 | 77.308 |  |

**Table 6** Improving hydrogen storage in MgH_2_ using 2D carbon-based composite catalysts

| **Composite Materials** | **Preparation method** | **Hydrogen adsorption** | **Hydrogen desorption** | **Remaining capacity/ cycles** | **Deh E_a_**  **(kJ/mol)** | **Ref.** |
| --- | --- | --- | --- | --- | --- | --- |
| 10 wt% TiH_2_@Gr | Ballmilling, Sonication | - 5.64 wt% at 300°C, 1.3 min. | - 6.77 wt% at 335°C. - Onset desorption at 204°C. | 97.6%/25 | 88.89 |  |
| 10 wt% FeOOH NDs@G | Hydrothermal | - 6.0 wt% at 200°C, 3.2 MPa, 1 h. | - 6.7 wt% at 325°C, 20 min. - Onset desorption at 229.8°C. | 98.5%/20 | 125.04 |  |
| 10 wt% Sc_2_O_3_/TiO_2_@Gn | Hydrothermal  ball milling | - 6.55 wt% at 300°C, 1 min | - 5.71 wt% at 300°C, 10 min - Onset desorption at 140°C | 95.3%/50 | 58.58 |  |
| 10 wt% Ni-Nb@rGO | Hydrothermal, Calcination | - 2.75 wt% at 75°C, 30 min, 3 MPa. - 4.5 wt% at 125°C, 10 min. | - 5.0 wt% at 245°C, 10 min. - Onset desorption at 198°C. | 93%/20 | 57.8 |  |
| 5 wt% Ti_2_C MXene | HF etching | - No mention. | - 7.5 wt% at 423°C. | / | 157.9 |  |
| 10 wt% 2V_2_C/Ti_3_C_2_ | HF etching | - 5.1 wt% at 40°C, 20 s, 6 MPa. | - 5.1 wt% at 225°C, 60 min. - Onset desorption at 180°C. | ≈100%/10 | 79.4 |  |
| N/S co-doped Nb_2_CT_x_ MXene | Calcination  ball milling | - 4.00 wt% at 30°C, 80 min, 30 bar | - 4.78 wt% at 225°C, 30 min | 80.77%/30 | 70.00 |  |
| 15 wt% In@Ti-MX | Self-assembly  ball milling | - 4.9 wt% at 200°C, 30 min - >3.9 wt% at 150°C, 3 h | - 5.25 wt% at 325°C, <10 min - Onset desorption at 290°C | / | 95.2 |  |
| 10 wt% (Ni, TiO_2_)@CN | MOF-derived | - 5.08 wt% at 40°C, 100 min. - 6.17 wt% at 125°C, 10 min. | - 5.85 wt% at 275°C, 10 min. - Onset desorption at 204°C. | / | 83.1 |  |

**Table 7** Improving hydrogen storage in MgH_2_ using 3D carbon-based composite catalysts

| **Composite Materials** | **Preparation method** | **Hydrogen adsorption** | **Hydrogen desorption** | **Remaining capacity/ cycles** | **Deh E_a_**  **(kJ/mol)** | **Ref.** |
| --- | --- | --- | --- | --- | --- | --- |
| 10 wt% Ni@NCS | Hydrothermal  Chemical reduction | - 5.7 wt% at 350°C, 8 min. - 4.2 wt% at 100°C, 60 min. | - 4.3 wt% at 350°C, 8 min. - Onset desorption at 246°C. | / | 75.6 |  |
| 5 wt% Ni@PHCNSs | Impregnation-reduction | - 6.2 wt% at 150°C, 250 s. - 5.3 wt% at 100°C, 600 s. | - 6.4 wt% at 275°C, 13 min. - Onset desorption at 190°C. | 94.1%/50 | 98 |  |
| 7 wt% Fe@HCS | Hydrothermal | - 5.6 wt% at 300°C, 55 s. | - 5.5 wt% at 300°C, 12 min. - Onset desorption at 225.9°C. | / | 84.97 |  |
| 10 wt% Pd_30_Ni_70_@CMK-3 | Solution impregnation | - 4.0 wt% at 70°C, 3 MPa, 5 h. - 5.8 wt% at 154°C, 100 min. | - 1.3 wt% at 100°C. - Onset desorption at 162°C. | / | 65.9 |  |
| CPF | Co-sintering | - 4.93 wt% at 200°C, 1 min. - 5.62 wt% at 150°C, 1 h. | - 5.67 wt% at 350°C, 1000 s. - Onset desorption at 284°C. | 99.1%/10 | 87.1 |  |
| 10 wt% FeNi@3DG | Sol-gel  Calcination | - 6.35 wt% at 300°C, 100 s. | - 5.13 wt% at 300°C, 500 s. - Onset desorption at ~ 189°C. | / | 83.8 |  |
| 10 wt% TM/C (Fe, Ni) | MOF-derived | - 6.8 wt% at 200°C, 60 s. - 5.7 wt% at 100°C, 1 h. | - 6.5 wt% at 323°C. - Onset desorption at 194°C. | 99.8%/10 | 77.308 |  |
| 10 wt% CoFe@C | MOF-derived | - 3.4 wt% at 100°C, 3600 s. | - 6.0 wt% at 300°C, 400 s. - Onset desorption at 175.9°C. | ≈100%/20 | 86.5 |  |
| 2.73 wt% Ni@C | Hydrothermal, Calcination | - 6.02 wt% at 150°C, 60 min. - 4.81 wt% at 100°C. | - 5.98 wt% at 257°C. - Onset desorption at 257°C. | ≈100%/10 | 77.6 |  |

**Table 8** Improving hydrogen storage performance in MgH_2_ using other types of compound catalysts

| **Composite Materials** | **Preparation method** | **Hydrogen adsorption** | **Hydrogen desorption** | **Remaining capacity/ cycles** | **Deh E_a_**  **(kJ/mol)** | **Ref.** |
| --- | --- | --- | --- | --- | --- | --- |
| 6 wt% Ni/Mo_2_N | Hydrothermal, Calcination | - 5.46 wt% at 150°C, 15 min. | - 5.92 wt% at 265°C, 30 min. - Onset desorption at 186.3°C. | 97.9%/10 | 76.35 |  |
| 7 wt% Ni/VN | Solvothermal | - 6.0 wt% at 150°C, 5 min. | - 5.6 wt% at 240°C, 1 h. - Onset desorption at 205°C. | 91.6%/10 | 78.07 |  |
| 5 wt% TiC | Hydrogen combustion | - 3.66 wt% at 100°C, 30 min. | - 2.5 wt% at 220°C, 30 min. | 98.3%/10 | 74.11 |  |
| 5 wt% ZrC | Hydrogen combustion | - 4.44 wt% at 150°C, 30 min. | - 4.7 wt% at 250°C, 30 min. | / | 90.37 |  |
| 5 wt% WC | Hydrogen combustion | - No mention. | - Onset desorption at ~ 216°C. | / | 88.29 |  |
| 7.5 wt% Ni/Mo_2_C@C | Pyrolysis | - No mention. | - 6.35 wt% at 300°C, 20 min. - Onset desorption at 240°C. | 97.8%/20 | 97.22 |  |
| 10 wt% Ni-MOF | Hydrothermal, Calcination | - No mention. | - 5.14 wt% at 300°C, 3 min. - Onset desorption at ~ 250°C. | 98.2%/10 | 100.05 |  |
| 10 wt% Ni-MOF@Pd | Wet-chemical synthesis | - 2.62 wt% at 100°C, 5 min. - 6.06 wt% at 150°C, 5 min. | - 6.5 wt% at 325°C, 30 min. - Onset desorption at 181°C. | / | 34.58 |  |
| 7 wt% MOFs-V | Hydrothermal, Calcination | - Begins at 60°C under 3200 kPa. | - 6.4 wt% at 300°C, 5 min. - Onset desorption at 190.6°C. | 98.9%/20 | 98.4 |  |
